# Resolving Complex Multiscale Structure of Magneto‐ and Electroactive Polymer Composites With an Ionic Liquid

**DOI:** 10.1002/adma.202516835

**Published:** 2026-04-01

**Authors:** Andrey Shibaev, Jon Maiz, Viktor Petrenko, Amaia Iturrospe, Josu Fernández Maestu, José María Porro, Mariano Barrado, Laura Casado, Joachim Kohlbrecher, Petr Shvets, Evgeny Modin, Ana Sofia Castro, Andrey Chuvilin, José María De Teresa, Daniela M. Correia, Arantxa Arbe, Senentxu Lanceros‐Méndez

**Affiliations:** ^1^ BCMaterials Basque Center for Materials Applications and Nanostructures Leioa Spain; ^2^ Centro de Física de Materiales (CFM‐MPC) CSIC‐EHU Donostia‐San Sebastián Spain; ^3^ IKERBASQUE ‐ Basque Foundation for Science Bilbao Spain; ^4^ Laboratorio de Microscopías Avanzadas (LMA) Universidad de Zaragoza Zaragoza Spain; ^5^ PSI Center for Neutron and Muon Sciences Villigen PSI Switzerland; ^6^ KönigsSystems UG Hamburg Germany; ^7^ CIC NanoGUNE Donostia‐San Sebastián Spain; ^8^ Centre of Chemistry University of Minho Braga Portugal; ^9^ Centre of Molecular and Environmental Biology University of Minho Braga Portugal; ^10^ Physics Center of Minho and Porto Universities (CF‐UM‐UP) and Laboratory of Physics for Materials and Emergent Technologies, LapMET University of Minho Braga Portugal; ^11^ Instituto de Nanociencia y Materiales de Aragón (INMA) CSIC‐Universidad de Zaragoza Zaragoza Spain

**Keywords:** electroactive polymers, ionic liquids, nanostructure, smart multifunctional composites

## Abstract

A multiscale understanding of the structure of ionogels – nanoparticle‐free polymer composites incorporating ionic liquids – is essential for enhancing their macroscopic functional properties and unlocking their potential in critical applications such as energy storage, sensing, and actuation. We establish a complete picture of the nano‐ and microstructuration of an ionic liquid within the matrix of a practically relevant electroactive copolymer poly(vinylidenefluoride‐*co*‐trifluoroethylene), by combining neutron scattering with cryogenic scanning electron tomography assisted by focused ion beam milling and cryogenic transmission electron microscopy with elemental analysis. We show that the ionic liquid is primarily located in the polymer amorphous phase and forms nanostructures with 10–12 nm size. It does not penetrate into the crystalline lamellae or the polymer amorphous phase confined between them, and it does not affect the polymer degree of crystallinity nor its complete crystallization in the highly electroactive β‐phase. Saturation of the unconfined amorphous phase with ionic liquid is identified as the key factor enabling high ionic conductivity while preserving mechanical integrity. At high ionic liquid concentrations, its excess microphase‐separates during the composite processing and accumulates in predominantly interconnected micrometer‐sized pores, providing the magnetoelectric response and further increasing the ionic conductivity.

## Graphical abstract

Multiscale ionic liquid segregation into nano‐sized domains in the unconfined amorphous polymer phase and into micron‐sized pores enables a coupled magneto‐ and electroactive responses.

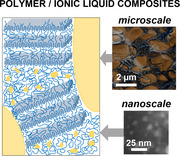

## 1 Introduction

“Smart” polymeric materials have a significant and increasing potential for a wide range of applications due to their ability to respond to external stimuli such as electric or magnetic fields, light, heat, and mechanical deformation, among others. A common route to impart such functional responses is the incorporation of nanoparticle fillers, including inorganic nanoparticles [1] or carbon‐based materials [2], into the polymer matrix. In the last years, an alternative nanoparticle‐free approach has been elaborated, which employs ionic liquids (ILs) instead of conventional fillers [3], providing so‐called ionogels [4, 5]. ILs are commonly defined as salts composed of an organic cation and an organic/inorganic anion that remain liquid at ambient conditions. They possess unique properties, including negligible vapor pressure, high chemical and thermal stability, flame retardancy, high ionic conductivity, and a broad electrochemical window [6]. ILs can be tailored through an extensive variety of cation‐anion combinations; therefore, they represent a highly adaptable platform for imparting specific functional responses to polymer matrices [7, 8, 9].

Composites that integrate ILs with various macromolecules [10, 11, 12] and, in particular, their combination with electroactive polymers [13] have gained attention for applications in sensors, actuators, and solid polymer electrolytes. The most commonly studied systems include poly(vinylidene fluoride) (PVDF) [14] and its copolymers such as poly(vinylidene fluoride‐*co*‐trifluoroethylene) (P(VDF‐TrFE)) [15, 16, 17]. Prior studies mainly addressed the macroscopic properties ‐ ionic conductivity, electrochemical actuation, magnetoelectric response, and others [3, 8, 13]. In particular, fluorinated polymer/ionic‐liquid composites based on P(VDF‐TrFE) have been widely investigated for electromechanical actuators and flexible sensors, where the combination of ionic conductivity and ferroelectric/piezoelectric response enables efficient conversion between mechanical and electrical signals. For example, PVDF‐based ionogel systems have been used for piezo‐ionic pressure sensors and wearable electronic skins, where ionic migration under mechanical stress generates measurable electrical signals [10]. IL‐containing fluoropolymer composites have been demonstrated as efficient materials for bending actuators, where ion migration inside the polymer matrix induces mechanical deformation under an applied electric field [8].

However, these properties strongly depend on the interaction of ILs with the polymer matrix, and in particular on how the IL is distributed within the polymer, especially among the crystalline and amorphous phases; and this structural aspect remains insufficiently clarified. Many ILs exhibit nanostructuring through amphiphilic aggregation even in bulk [18], and these supramolecular arrangements are further altered by confinement [19]. Semicrystalline polymers impart an additional level of complexity through a phase coexistence (crystalline phase; amorphous phase confined between the crystalline lamellae; and unconfined amorphous phase [20]). Determining how ILs partition within each region is essential but currently unresolved. Small‐angle X‐ray/neutron scattering (SAXS and SANS) and cryogenic electron microscopy are unique methods to explore the structure at the nano‐ and micro‐levels, and are well suited for this purpose. Moreover, despite its potential, cryogenic focused ion beam (cryo‐FIB) tomography has not been employed in the investigation of the native structure of ionogels.

Recent research has explored magnetic ILs within PVDF‐based matrices as an alternative to magnetic nanoparticle fillers [21, 22]. Incorporation of ILs such as 1‐alkyl‐3‐methylimidazolium tetrachloroferrates ([C_n_mim][FeCl_4_], n = 2,4,8,14) or bis(1‐ethyl‐3‐methylimidazolium) tetrathiocyanatocobaltate ([Emim]_2_[Co(SCN)_4_]) can alter PVDF crystallization, promoting electroactive β or γ phases [23, 24, 25]. Blends of P(VDF‐TrFE) with 1‐butyl‐3‐methylimidazolium tetrachloroferrate ([Bmim][FeCl_4_]) show a notable magneto‐ionic response, exhibiting electric potential generation in the external magnetic field with a magnetoelectric coefficient up to 10 V·cm^−1^·Oe^−1^ at low magnetic fields (0.5 Oe) [21, 26], and change their resistance in the presence of water vapor [27]. Despite these promising macroscopic effects, the distribution and nanoscale organization of magnetic ILs remain largely unexplored, and only a few studies have reported IL nanostructuration in these systems [28]. A systematic, multiscale structural analysis is still missing.

With these ideas in mind, this study aims to investigate the micro‐ and nanostructure of P(VDF‐TrFE) composites containing different amounts of the widely used magnetic IL [Bmim][FeCl_4_], and to correlate this structure with their functional properties. To properly face this problem, we apply a novel and exhaustive methodology that combines a suite of scattering, microscopy, and tomography techniques, enabling the structural characterization at different relevant length scales. The key advancements of this work are as follows: (1) quantitative separation of the contributions to neutron scattering, revealing that the IL forms nanostructures exclusively within the un‐confined amorphous phase; (2) direct visualization of these nanostructures in the native state by cryogenic high‐angle annular dark field scanning transmission electron microscopy (cryo‐HAADF‐STEM) with elemental amalysis; (3) confirmation and visualization of IL accumulation inside micron‐sized pores using cryogenic scanning electron microscopy (cryo‐SEM) with spectroscopic elemental analysis; (4) 3D reconstruction of the native pore network via layer‐by‐layer cryo‐FIB tomography, revealing the interconnections between the micropores; (5) combined scattering/microscopy analysis enabling quantitative determination of IL partitioning between pores and matrix (which may be applied for various systems based only on room‐temperature SEM analysis); 6) demonstration of the interrelation between the micro‐/nanostructure and macroscopic properties (ionic conductivity, mechanical properties, magnetoelectric performance and magnetomechanical bending). These findings provide essential insights linking multiscale structure (from micro‐ to nanoscale) to macroscopic behavior, thereby advancing the design of polymer/IL composites for sensing and actuation applications.

## 2 Results and Discussion

### 2.1 Crystalline Morphology

**FIGURE 1:**
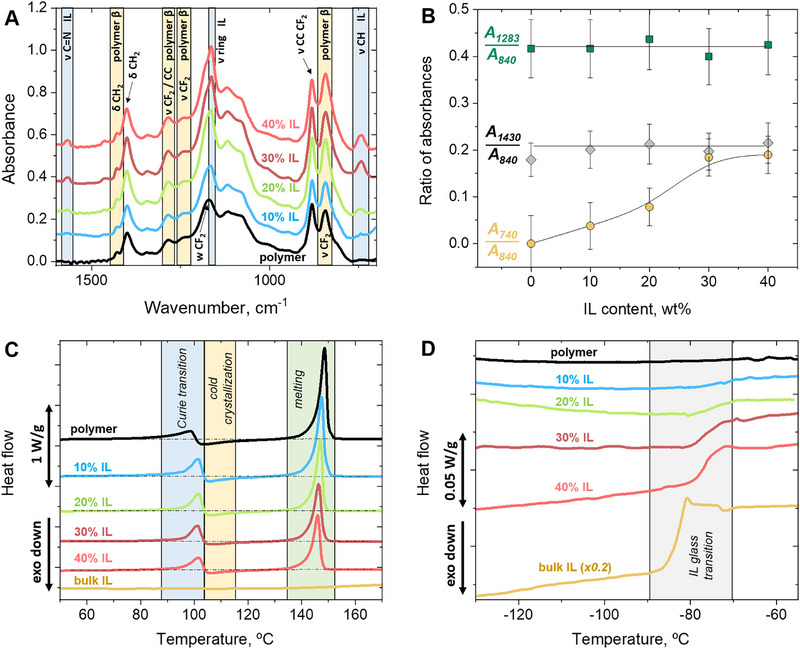
Crystalline morphology and phase transitions in P(VDF‐TrFE) / [Bmim][FeCl_4_] composites and their components: (A) ATR‐FTIR spectra at varying IL content (offset for clarity) showing various molecular vibrational bands; (B) Ratios of baseline‐corrected infrared absorbances at selected wavenumbers: A_1283_/A_840_ and A_1430_/A_840_ (polymer bands) and A_740_/A_840_ (ratio of IL band to polymer band); the lines serve to guide the eye; (C) DSC first heating profiles, highlighting Curie transition, cold crystallization and melting; (D) Second heating profile, showing glass transition of the IL.

First, the influence of the [Bmim][FeCl_4_] on the properties of the P(VDF‐TrFE) crystalline phase was evaluated using attenuated total reflectance Fourier‐transform infrared spectroscopy (ATR‐FTIR) and differential scanning calorimetry (DSC). Several distinct peaks associated with the polar and highly electroactive polymer β‐phase (resulting from all‐trans conformation of the polymer chains) [29] appear in the FTIR spectra of both the neat polymer and composites (Figure 1A). These include the absorption bands at 1283 and 1430 cm^−1^ attributed to the CF_2_ stretching vibrations and CH_2_ bending vibrations, respectively [30], along with an intense CF_2_ stretching vibration peak at 840 cm^−1^ known as a signature of the β‐phase [31]. The lack of strong bands at 766 and 976 cm^−^^1^ indicates the absence of non‐polar α‐phase (formed by polymer chains in trans‐gauche‐trans‐gauche conformations). Thus, FTIR confirms that P(VDF‐TrFE) crystallizes in the electroactive β‐phase both with and without [Bmim][FeCl_4_], aligning with the data from similar composites prepared at different conditions [22]. The peaks at 740 cm^−1^ (C─H vibrations), 1164 cm^−1^ (ring stretching), and 1564 cm^−1^ (C═N stretching) are attributed to the Bmim^+^ cation of the IL [32, 33]. The ratio of peak intensities at 740 (IL) and 840 cm^−1^ (polymer) increases with rising IL content (Figure 1B). At the same time, the ratios of various polymer peak intensities remain unchanged upon IL addition, suggesting that the crystalline structure of the polymer matrix is not altered.

In the DSC first heating profiles, a ferroelectric‐to‐paraelectric Curie transition is observed, followed by a minor exothermic peak due to cold crystallization, and finally, melting (Figure 1C; transition temperatures are summarized in Table S1). The Curie temperatures (*T_Curie_*) both in the absence and in the presence of IL are close to the reported values of 102°C–110°C [30, 34]. Cold crystallization, rarely observed for P(VDF‐TrFE), follows the Curie transition. This effect likely results from defects in the polymer crystalline phase formed due to a rapid solvent (acetone) evaporation during composite preparation. At the Curie transition, polymer chains reorganize, facilitating additional crystallization. However, cold crystallization disappears in the second heating cycle after melting (Figure S1), supporting the hypothesis that this effect arises from the specific conditions during the composites´ preparation. The overall degrees of crystallinity (*χ_c_*) were calculated from DSC data with the equation [31]

(1) $$\chi_{c}=\frac{\Delta H_{m}-\Delta H_{cc}}{m{\%}_{pol}\;\;\Delta H_{100}}100\%$$

where Δ*H_m_* and Δ*H_cc_* represent the enthalpies of melting and cold crystallization, respectively, Δ*H_100_* = 103.4 Jg^−1^ is the melting enthalpy of the 100% crystalline PVDF in the β‐phase [31], and *m%_pol_* = *φ_pol_ · ρ_pol_* is the polymer mass fraction in the composite (*φ_pol_* is the polymer volume fraction, and *ρ_pol_* is the polymer density). *χ_c_* remains almost constant within the range of 22%–23% regardless of [Bmim][FeCl_4_] concentration (Table S1). This suggests that the IL predominantly resides in the amorphous polymer phase not integrated within the lamellar stacks due to the partial miscibility of the components [35, 36, 37]. However, it does not enter the amorphous phase confined between the crystalline lamellae [38, 39].

Thus, IR spectroscopy and calorimetry data confirm that the crystalline structure of P(VDF‐TrFE) remains unaffected by [Bmim][FeCl_4_] incorporation. However, the IL undergoes a glass transition at concentrations above 20 wt.%, manifested as a pronounced specific heat step at the DSC curves (Figure 1D). This is similar to the IL bulk behavior ‐ it exhibits a bulk glass transition at *T*_g_ = −85°C. To further investigate the molecular origins of these phenomena, structural studies using electron microscopy and SANS/SAXS techniques were conducted.

### 2.2 Microstructure

**FIGURE 2:**
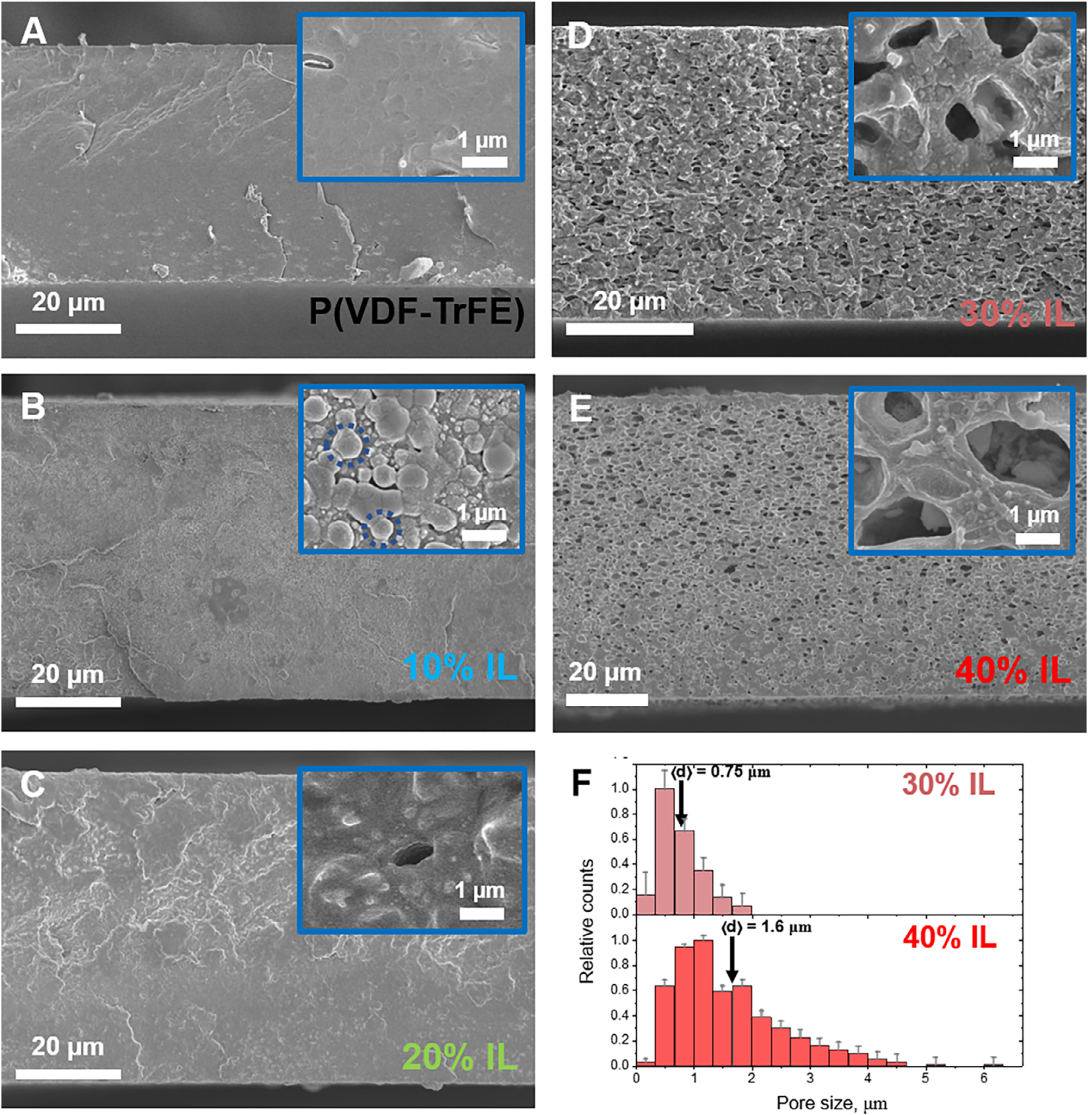
Cross‐section SEM micrographs of neat P(VDF‐TrFE) film (A) and its composites with various amounts of [Bmim][FeCl_4_]: 10 (B), 20 (C), 30 (D), and 40 wt.% (E). The insets highlight magnified regions of the cross‐sections. Size distributions of pores in the samples with 30 and 40 wt.% [Bmim][FeCl_4_] (F).

To assess the effect of IL on the composites’ structure at the microscale, SEM analysis was employed, which shows a dramatic structural change occurring above a critical concentration of 20 wt.% IL (Figure 2). The neat polymer film exhibits a homogeneous morphology at the microscale (Figure 2A). For a small IL amount (10 wt.%), micron‐sized spherical objects are observed (Figure 2B), similar to the ones reported previously [28]. They appear most likely due to the minimization of surface energy of the growing polymer/IL structures during polymer crystallization. For 20 wt.% IL, the morphology is similar, and some isolated micropores are present (Figure 2C). At 30 and 40 wt.% [Bmim][FeCl_4_], the microstructure changes drastically – many micropores are seen (Figure 2D,E). The micropore size significantly increases with the rise of IL concentration (Figure 2F). A similar behavior was previously reported for P(VDF‐TrFE)/[Bmim][FeCl_4_] composites prepared from N,N‐dimethylformamide (DMF) solutions, and the formation of micropores was attributed to a microphase separation between P(VDF‐TrFE) and the IL/DMF mixture during the film preparation, followed by the IL occupying free spaces (pores) left by the solvent [21]. However, the pores appear darker than the surrounding matrix at the SEM micrographs, suggesting that they are empty, most likely due to the procedure of SEM specimen preparation (implying de‐freezing of the sample) which results in leaking of the IL out of the pores (see Experimental Section). Consequently, direct evidence confirming the presence of IL within these micropores in the composites´ native state, which is practically important for the functional properties of the composites, including a magneto‐ionic response [21], has yet to be presented.

**FIGURE 3:**
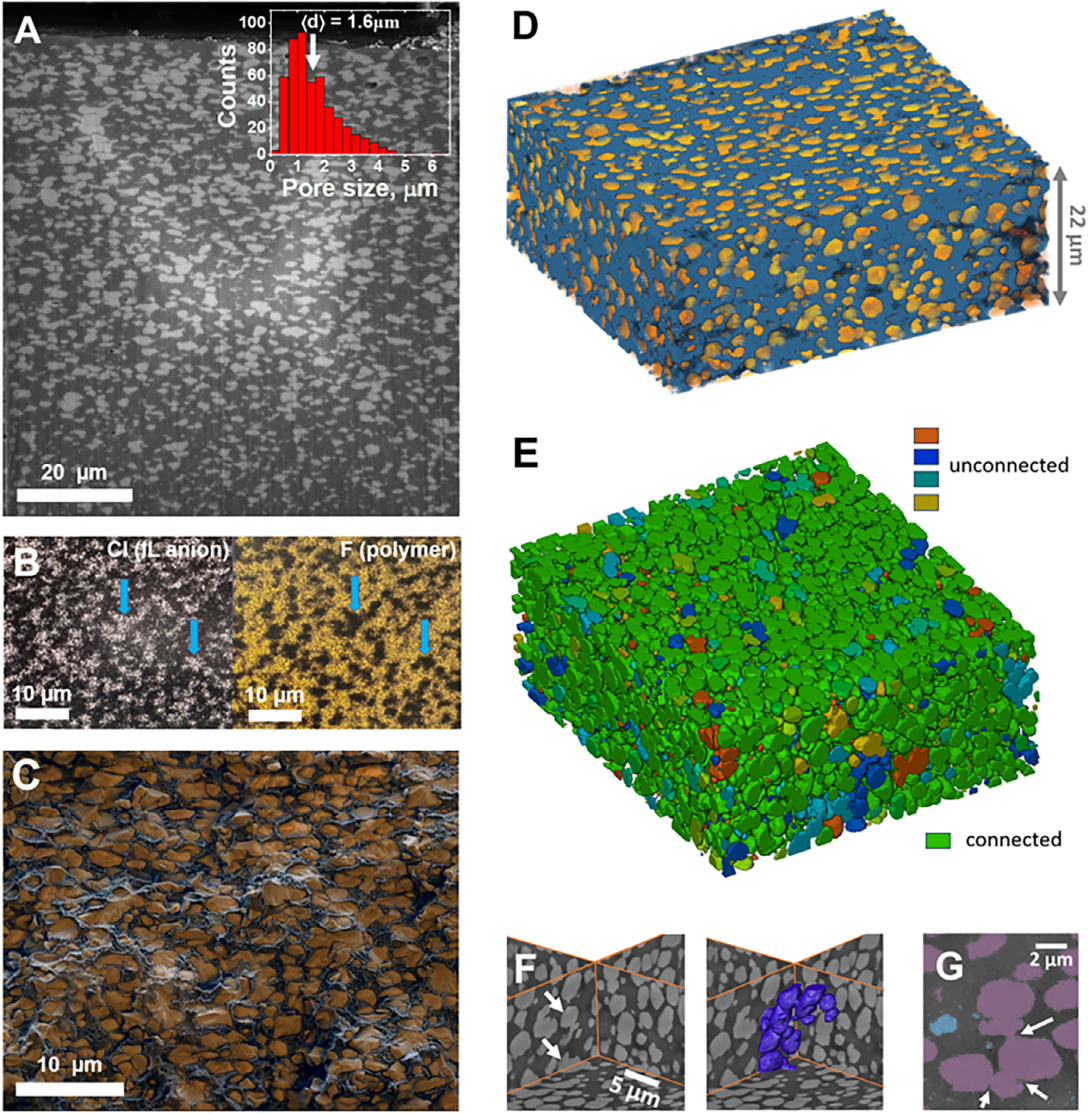
Cryo‐FIB/SEM imaging and tomography revealing the microstructure of the P(VDF‐TrFE) composite with 40 wt.% [Bmim][FeCl_4_]: (A) Cross‐section micrograph obtained by FIB milling (inset shows the size distribution of the lighter areas in the micrograph ‐ pores); (B) Energy‐dispersive X‐ray (EDX) elemental maps of Cl and F in a selected region of a freeze‐fractured sample; (C) False color reconstruction of the composite microstructure obtained by overlapping signals of the surface relief (secondary electrons, SE) and of the elemental contrast (backscattered electrons, BSE) from a freeze‐fractured sample, showing the polymer matrix in black and IL in the micropores in orange; (D) 3D reconstruction of the microstructure showing the polymer matrix in blue and IL in the micropores in orange, based on layer‐by‐layer cryo‐FIB milling and cryo‐SEM imaging; (E) 3D reconstruction, where all micropores interconnected with each other are colored in green, unconnected pores are colored differently, and the polymer matrix is not shown; (F) perpendicular slices of the 3D reconstruction showing individual pores (left), and part of the 3D reconstruction highlighting how the pores, marked by arrows on the left, are interconnected (right); (G) Small region of the reconstruction slice, showing pores in violet, polymer matrix in dark grey, and highlighting “bridges” that connect the pores by white arrows.

To determine the distribution and location of the IL within the polymer matrix, cryo‐FIB/SEM combined with elemental analysis was conducted. Electron microscopy at cryogenic conditions is advantageous for this purpose, as it allows preserving the intact structure of the IL within the composite. Representative cross‐section images of the microporous samples containing 40 (Figure 3A) and 30 wt.% IL (Figure S2) show the micron‐sized objects within the polymer matrix. These are micropores which remain filled with the IL under the cryogenic preparation conditions. This is confirmed by the fact that they appear brighter than the surrounding matrix, because the IL anion contains iron, giving strong compositional contrast in the backscattered electron (BSE) signal. Interestingly, the movement of the IL inside the micropores was directly observed in cryo‐SEM (Video S1). Presumably, local heating due to beam exposure increased the mobility of the IL inside the micropores, resulting in its displacement due to the accumulation of charge.

The presence of [Bmim][FeCl_4_] in the micropores is further evidenced by EDX mapping/spectroscopy (Figure 3B; Figure S2), showing that the microdomains rich in fluorine (F from the polymer) lack chlorine (Cl from the IL anion), and vice versa, confirming a partial microphase separation between the polymer and the IL. Notably, the IL´s cations and anions must reside within the same microdomains, as their separation at the microscale would lead to an unfavorable increase in electrostatic energy. The overlap of the secondary electrons signal (relief) and the BSE signal (elemental contrast) allows restoring the composite´s microstructure and confirms that the polymer and IL are indeed microphase separated, with the IL primarily residing in micrometer‐sized pores within the polymer matrix (Figure 3C). The pores are characterized by a slightly elongated shape with an aspect ratio of 1.8, and their longer axes are preferentially oriented parallel to the film surface. This is presumably due to the film preparation procedure: the IL/acetone bubbles are first microseparated from the polymer matrix, but then the solvent continues to evaporate, and the film dries, reducing its thickness. This may result in a slight compression of pores in the vertical direction, which cannot fully restore the spherical shape due to the high viscosity of the medium.

For the practical application of these composites in areas such as actuators, it is essential to determine whether the IL‐filled domains are interconnected, as the actuation relies on charge movement and microseparation [26]. To investigate this, cryo‐FIB/SEM tomography was performed, that enables reconstruction of the 3D structure of the IL domains and analysis of their connectivity. The 3D reconstruction of a certain composite volume is shown in Figure 3D and in Video S2. In Figure 3E, all the pores connected to each other are shown with a single color (green), while the isolated clusters are colored differently. The majority of the pores are confirmed to be interconnected, forming a percolated network. This is verified by a hand‐mode segmentation of several IL domains. In Figure 3F (left), two micropores, which seem not to be connected, are marked by arrows. However, a part of the 3D reconstruction (Figure 3F, right) reveals that these pores are connected via several others. At the 2D slices of the reconstruction, the connections between the adjacent pores are clearly observed (Figure 3G). At the same time, only a small fraction of the IL clusters (5 vol% of all the IL‐rich microdomains) is isolated without direct contact with pores located far from them (Figure S3). A percolated network of the IL‐filled micropores contributes to the macroscopic properties of the composites (e.g., electrical conductivity), as shown in the following sections.

Thus, cryo‐FIB/SEM/tomography combined with EDX spectroscopy reveals a critical IL content of 20 wt.%, separating two distinct structural regimes. At high concentrations (30 and 40 wt.%), the IL is partially microphase‐separated from the polymer and is located in the micron‐sized pores. However, some fraction of the IL is embedded within the polymer microphase, as suggested above by FTIR and DSC. This most probably occurs at all the concentrations, including 10 and 20 wt.% IL composites with almost no micropores (Figure 2B,C). In non‐porous composites, fully organic ILs have been reported to reside in the amorphous regions of PVDF [35, 36]. A similar hypothesis was proposed for [Bmim][FeCl_4_] in the work [22], and qualitative evidence for the location of [Emim]_2_[Co(SCN)_4_] within the PVDF matrix was put forward in the publication [25]. To investigate this nanoscale structural organization, SANS, SAXS, and cryo‐HAADF‐STEM were employed in the present work.

### 2.3 Nanostructure

**FIGURE 4:**
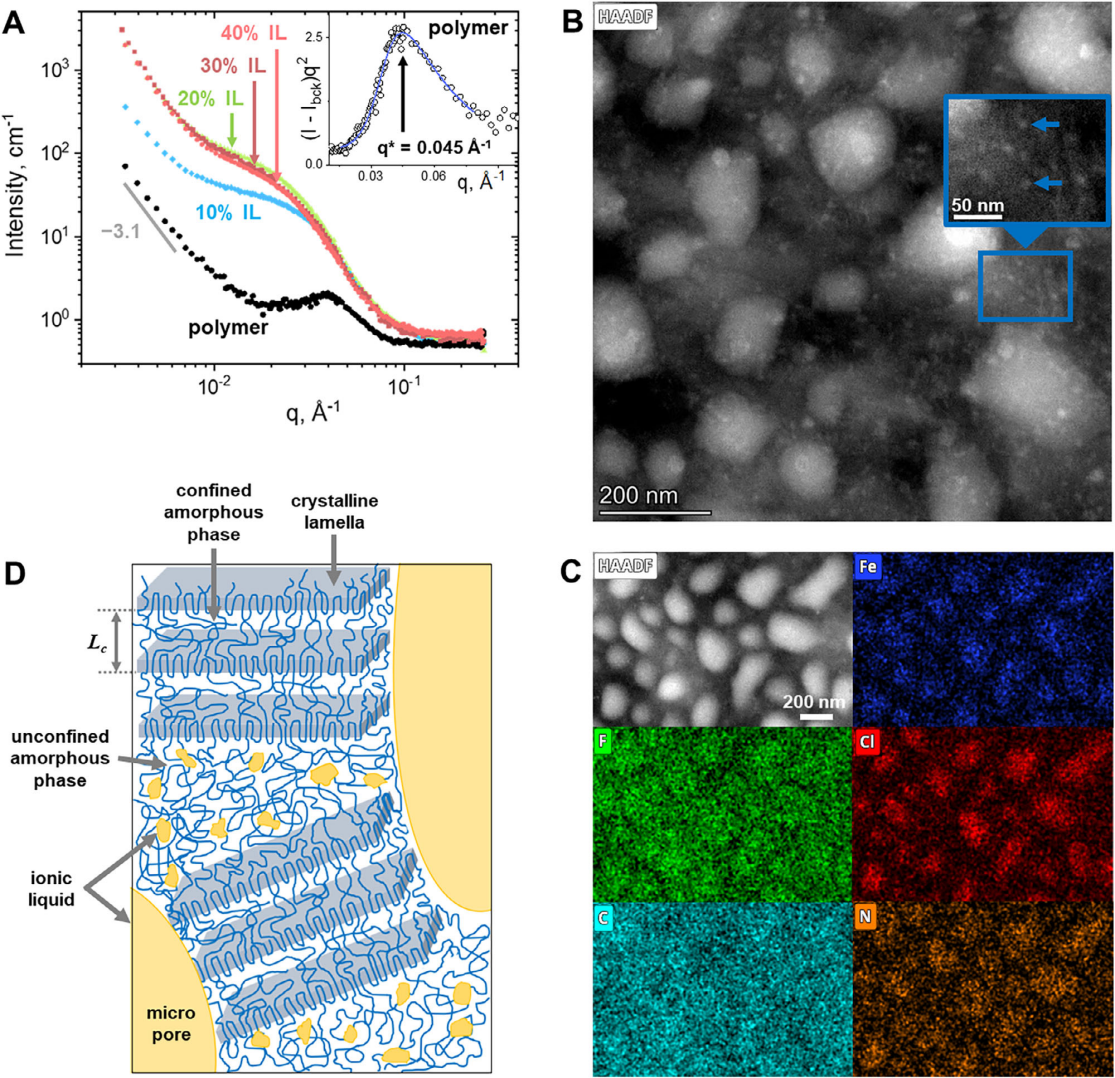
Neutron scattering and cryo‐HAADF‐STEM/EDX data revealing the nanostructure of the polymer/IL composites: (A) SANS curves for P(VDF‐TrFE) composites with different concentrations of [Bmim][FeCl_4_] (see fits by Equation (2) in Figure S4). Inset corresponds to the Lorentz‐corrected plot ((*I‐I_bck_)q* ^2^ vs *q*, where *I_bck_* is the incoherent background scattering) for the neat P(VDF‐TrFE). Blue line is a fit by an asymmetric double sigmoidal function. (B,C) Cryo‐HAADF/STEM micrographs and the corresponding elemental maps of the composite with 5 wt.% [Bmim][FeCl_4_]; a region with 10‐nm nanostructures is magnified in Figure (B). (D) Schematic representation of the micro‐ and nanostructure of the P(VDF‐TrFE) composites with 30 or 40 wt.% [Bmim][FeCl_4_], where the IL is located both in the micropores and in the amorphous polymer phase.

SANS scattered intensities *I*(*q*) for P(VDF‐TrFE) and its composites with various amounts of IL are shown in Figure 4A, indicating the development of a nanostructure with IL content increasing from 0 to 20 wt.%. The scattering curve for neat polymer exhibits a profile characteristic of PVDF and other semi‐crystalline polymers, showing a power‐law decay at low *q* (bigger sizes), which is a result of Porod scattering from the surface of large microstructures (e.g., assemblies of crystalline lamellae) [40]. A structure peak at intermediate *q* corresponds to the parallel stacking of the crystalline lamellae, interspersed with the confined amorphous phase layers [41, 42]. It arises from the difference in the neutron scattering length densities (SLDs) of the crystalline and amorphous phases (Table S2). From the peak position (q*) in the Lorentz‐corrected plot (*Iq* ^2^ vs. *q*, inset in Figure 4A), the lamellar long period is determined as *L_c_* = 2π/q* = 14 nm, which is close to the values reported previously [43, 44].

A significant increase in scattered intensity is observed when the IL is embedded in the composites (Figure 4A). Several factors could contribute to this effect. First, as discussed earlier, at least a portion of the IL resides in the pores within the polymer matrix for 30 and 40% IL content. However, these pores are micro‐sized, much larger than the size range probed by SANS (∼ 1–100 nm). Thus, the IL in these pores can be considered bulk‐like and contributes only to the increase of Porod (surface) scattering from the pores´ interfaces identified as an intensity upturn at low *q* values (Figure 4A). Notably, the bulk IL does not exhibit distinct SANS or SAXS structural patterns, showing only incoherent scattering in the investigated *q* range. In addition to the micropores, scattering from the interface of other large objects (e.g. assemblies of polymer crystalline lamellae) also contributes to the Porod scattering in the low *q* range, as seen for the neat polymer [45].

Second, scattering from the polymer matrix contributes to the SANS signal of the composites. As shown above, the polymer crystalline structure is unaffected by the IL. This is further supported by the SANS data. Indeed, [Bmim][FeCl_4_] has an SLD lower than the ones of both amorphous and crystalline polymer phases (Table S2). Therefore, if it entered the confined amorphous phase, it would lower its SLD relative to the crystalline lamellae, increasing the contrast and enhancing the intensity and sharpness of the structural peak. This effect is not observed in the SANS curves; instead, the peak is progressively hindered by scattered intensity arising from different scattering contributions (Figure 4A). For some organic ILs, incorporation into the rigid amorphous phase between crystallites has been reported to reduce the lamellar thickness due to the strong IL‐polymer interactions [35]. In our case, a weaker affinity between [Bmim][FeCl_4_] and the polymer explains that the IL does not integrate into the lamellar stacks. Instead, it is excluded from the growing crystals, which is further amplified by rapid crystal growth due to fast solvent evaporation (as indicated by the presence of cold crystallization, Figure 1C). Therefore, scattering from the polymer matrix is assumed to be the same for all the composites and equal to the neat polymer scattering (taking into account the polymer volume fraction).

Third, the polymer and the IL are not completely microphase separated, and some IL fraction resides in the amorphous phase not confined within the crystallites. It is likely that the IL forms nanostructures in this phase, contributing to the enhanced SANS signal.

Consequently, the scattered intensity in the composites arises from three sources: (1) scattering from the surface of large objects *I_Porod_* (giving rise to the Porod exponent at low *q* values); (2) scattering from the polymer crystalline lamellar stacks normalized by the polymer volume fraction *φ_pol_I_pol_* (*I_pol_* remains the same across all samples); (3) scattering from IL nano‐aggregates within the unconfined amorphous phase *I_IL_*. The total intensity can thus be expressed by the following formula:

(2) $$\begin{array}{ccc} I\left(q\right) & = & I_{Porod}+\varphi_{pol}I_{pol}+I_{IL}=A_{Porod}q^{-P}\\ & & +\varphi_{pol}\;A_{pol}\frac{S_{pol}\left(q\right)}{q^{2}}{\left(SLD_{cryst}-SLD_{am}\right)}^{2}\\ & & +\varphi_{IL}\;A_{IL}\;P_{IL}\left(q\right)\;V_{IL}\;{\left(SLD_{IL}-SLD_{am}\right)}^{2} \end{array}$$

Here *A_Porod_*, *A_pol_*, and *A_IL_* are the coefficients showing the contributions of each of the above‐mentioned sources to scattering; *P* is the Porod exponent at low scattering vectors; *S_pol_(q)* is the structure factor accounting for the lamellar organization and containing the structure peak of the neat polymer scattering; *P_IL_(q)* is the form‐factor of the IL accumulations within the unconfined amorphous phase; *V_IL_* is the volume of one IL accumulation; *φ_IL_* = 1 ‐ *φ_pol_* is the volume fraction of the IL; and *SLD_cryst_*, *SLD_am_* and *SLD_IL_* are the corresponding scattering length densities of the crystalline lamellae, amorphous phase and the IL, respectively (Table S2). The *q^−2^* factor in the second term accounts for the form‐factor of the crystalline lamellae, which are locally “flat” [46]. It allows extracting the lamellae long period from the Lorentz‐corrected plot (Figure 4A), since multiplication of the intensity by *q^2^* “eliminates” the *q*‐dependence of the lamella´s form‐factor [47].

Fitting of the Porod exponent at low *q*, and subtracting the Porod scattering and the neat P(VDF‐TrFE) scattering (first and second terms of Equation 2) from the composite SANS curves isolates the contribution from the IL aggregates (third term). This is fitted with the form‐factor of polydisperse spheres (the model is limited to this form‐factor in accordance with the cryo‐HAADF‐STEM results presented below). Figure S4 shows the decomposition of the fits at various IL concentrations into these three contributions, and the total fits of the scattering curves; the obtained values of fit parameters are summarized in Table S3. Notably, the third term ‐ scattering of the IL accumulations ‐ closely resembles the total scattering curves in the intermediate *q* range (0.015–0.05 Å^−1^, which corresponds to size range ∼ 2π/*q* = 40–10 nm), indicating that the IL contributes significantly to the scattering signal of the composites. The diameter of the IL accumulations *d_IL_* is in the range of 10‐12 nm and slightly increases with the IL concentration (Table S3). Considering a volume of a single [Bmim][FeCl_4_] molecule *V_1_* = *M* / (*N_A_ · ρ_IL_*) = 0.41 nm^3^ (*M* and *ρ_IL_* are the molecular weight and density of [Bmim][FeCl_4_]; *N_A_* is Avogadro's number) and the volume of one IL accumulation *V_IL_* = 3/2π*d_IL_* ^3^ ≈ 820 nm^3^, the number of IL molecules in one nano‐accumulation is *V_IL_* / *V_1_* ∼ 2000. Previously, similar nano‐accumulations were evidenced for fully organic ILs embedded in PVDF‐based polymers. For instance, 1‐vinyl‐3‐butylimidazolium bis(trifluoromethylsulfonyl)imide was shown to form 10–20 nm droplet‐like structures in poly(vinylidene fluoride‐co‐hexafluoropropylene) [48]. Elongated nanodomains of amphiphilic IL octadecyltriphenylphosphonium iodide were observed in poly(vinylidene fluoride‐co‐chlorotrifluoroethylene) [36].

Cryo‐HAADF‐STEM/tomography allowed direct visualization of the IL nanostructures within the polymer matrix (Figure 4B), complementing and supporting the SANS data. For these experiments, a low IL concentration of 5 wt.% was chosen, in order to separate spatially the IL nanostructures. HAADF imaging utilizes Z‐contrast, highlighting IL accumulation rich in iron. IL nanostructures of two sizes are seen at the micrographs–approximately 10 and 100–200 nm. The smaller nanostructures, visualized in the inset of Figure 4B, are most likely the ones detected by SANS. The bigger nanostructures fall out of the range covered by SANS. EDX mapping (Figure 4C) supports these findings, showing that the brighter objects at the HAADF‐STEM images are rich in Fe, Cl, and N, which are present in the IL. At the same time, the C map is much more uniform, because both the polymer and the IL contain it. The F map proves that there is no phase separation between the components (at low IL concentration), giving additional proof to the SEM/cryo‐SEM analysis presented above. Finally, tomographic reconstruction (Video S3) demonstrates that the IL nanoaccumulations are not interconnected at low IL content, contrary to the case of 40 wt.% IL with a network of interconnected micropores (Figure 3E).

The hypothesis of the formation of IL nanostructures is further supported by temperature‐dependent SAXS measurements (Figure S5). At 140°C (near the melting onset) and above 160°C (fully molten state), the scattered intensity progressively decreases due to the enhanced IL‐polymer mixing at elevated temperatures, when the polymer matrix melts. At 160°C, where the composite is completely molten (Figure 1C), a faint structural feature at *q* ≈ 0.02 Å ^−1^ persists, with higher intensity than at 200°C, suggesting that the IL nanostructures still exist just above the melting temperature. By 200°C, all structural features vanish, leaving only a power‐law decay with a slope *I* ∼ *q* ^−4^, characteristic of the thermal composition fluctuations observed in homogeneous polymer melts [49].

To summarize, at 10 and 20 wt.% [Bmim][FeCl_4_], SANS intensity increases with IL concentration (Figure 4A), indicating that the IL forms a higher number of nanostructures when its content is increased. On the contrary, the SANS scattering curves for 20, 30, and 40 wt.% IL nearly overlap, suggesting that the nanostructures remain unchanged beyond 20 wt.%. Thus, decomposition of the neutron scattering curves into several contributions, combined with cryo‐HAADF‐STEM data, reveals that the IL forms 10–12 nm‐sized nanodomains within the unconfined amorphous phase of the polymer matrix, which is the main reason for the upturn of the scattered intensity in the intermediate range of scattering vectors.

### 2.4 Quantitative Analysis of the IL Distribution

A combined analysis of SANS and electron microscopy data provides a deeper insight into the IL distribution within the polymer matrix. At 30 and 40 wt.% IL, micrometer‐sized IL‐filled pores are present, implying that 20 wt.% is the maximum [Bmim][FeCl_4_] concentration that can be incorporated into the amorphous polymer phase. Therefore, any excess of IL beyond 20 wt.% phase‐separates into the micropores.

For the 40 wt.% IL composite, the relative pore surface area averaged over the whole 2D cross‐section of the film (for instance, cross‐section in Figure 3A) S_pores_/S_tot_ equals to 0.3, as calculated by the image processing using automatic pore segmentation with a brightness threshold. Considering a simplified case of the spherical pores and implying a geometrical equation linking the sphere cross‐sectional area to its volume, the pore volume fraction is estimated as:

(3) $$\frac{V_{pores}}{V_{tot}}\approx \frac{4}{3\sqrt{\pi }}{\left(\alpha \frac{S_{pores}}{S_{tot}}\right)}^{3/2}∼\;0.23$$

Here α = 1.5 is a coefficient that accounts for the fact that not all the pores are cross‐sectioned in the middle of their radius at the 2D image, but may be located “deeper” or “shallower,” which results in an underestimation of their cross‐sectional area. At a total 40 wt.% of IL, half of it resides in the micropores and half is located in the amorphous polymer phase. For this case, a theoretical calculation gives *V_pores _*/ *V_tot_* = 0.24 (Section S7), which is in excellent agreement with the experimental value of 0.23 obtained from Equation (3). If all the IL was confined in the pores with none in the polymer phase, this theoretical value would increase to *V_pores_* / *V_tot_* = 0.47 (Supporting Information), not matching the experimental data. These estimates confirm that the IL is distributed between the micropores and the polymer matrix.

This conclusion is directly supported by the cryo‐SEM EDX spectroscopy (Figure S2). The amount of Cl is significantly higher in the micropore than in the surrounding polymer matrix, indicating that the micropore is filled with the IL. At the same time, the polymer matrix also contains a certain IL amount, and its concentration semi‐quantitatively coincides with the above calculations (for the 30 wt.% IL composite, it is assumed that ∼20 wt.% is embedded in the polymer matrix, while ∼10 wt.% phase separates into the micropores).

Combined electron microscopy and SANS structural investigations thus reveal the multiscale structure of the P(VDF‐TrFE) composites with [Bmim][FeCl_4_], schematically depicted in Figure 4D. The IL does not penetrate into the crystalline lamellar packing. At all the investigated IL concentrations, it enters the unconfined amorphous phase and forms nano‐sized accumulations with rather similar average sizes. Finally, above a certain maximum IL concentration (around 20 wt.%), the amorphous phase is saturated with the IL, and its excess micro‐segregates into the micropores.

Finally, electron microscopy allows a more detailed analysis of the IL distribution within the 40 wt.% IL composite. Figures 2E and 3A show that the distribution of the micropores with depth is not uniform: e.g., they are almost absent at the bottom of the film. Depth profiling of the IL concentration with EDX spectroscopy (Section S8) shows that the IL is present at all depths, which is important for the macroscopic properties, for instance, conductivity. Layers rich in micropores also contain higher IL concentration (Figure S6). At the same time, the depth profile of IL concentration for the 10 wt.% IL composite (with no micropores) is uniform (Figure S6D). These data provide additional confirmation that the pores are filled with the IL, and their presence contributes to the local IL content within the composites.

The IL distribution observed in this study differs from that reported in the work [21], where micron‐sized pores were observed at 10, 20 and 40 wt.% IL (though rare and small at 10 wt.%). The discrepancy is likely due to a different procedure of sample preparation. In the work of Correia et al. [21], composites were prepared by evaporating DMF at 210°C (above the P(VDF‐TrFE) melting temperature) and cooling down to room temperature, allowing the IL sufficient time to phase‐separate from the polymer. In contrast, our composites were prepared by fast acetone evaporation at 50°C (below the polymer melting temperature), meaning that the solvent evaporation and polymer crystallization occurred simultaneously, leading to greater IL entrapment within the polymer phase. Therefore, changing the solvent evaporation rate allows tuning the micro‐ and nanostructure of the composites, which is important to adjust their functional response.

### 2.5 Macroscopic Properties

The observed micro‐ and nanostructure of the polymer/IL composites is a key factor determining their macroscopic characteristics. The composites´ structure governs the ionic conductivity, which is important for the magneto‐ionic response, underlying the bending actuator performance [21], application of the polymer/IL composites as piezo‐ionic sensors [50, 51], and as materials for batteries [52]. Frequency dependences of the real part of conductivity σ´(*f*) are shown in Figure S7 for the samples with different amounts of IL, as well as for the polymer and IL taken alone. Since neat P(VDF‐TrFE) is a dielectric polymer, its conductivity is extremely low in the whole frequency range (Figure S7A). This behavior is consistent with the previous reports [53].

A 10 wt.% concentration of IL induces a drastic increase in low‐frequency conductivity and appearance of a plateau (Figure S7B), which is evidently due to the ionic conductivity imparted by the IL entrapped within the amorphous phase of the polymer matrix. However, the high‐frequency conductivity remains almost equal to the values for the neat polymer at all temperatures. At 20 wt.% IL, the conductivity is higher for the composites as compared to the neat polymer in a much wider frequency range, and a well‐developed plateau is observed above −53°C (Figure S7C). This result shows that the enrichment of the amorphous phase by IL (evidenced above by electron microscopy and SANS) is a crucial factor determining the conductivity. Note that the micropores are almost absent in the 20% IL sample, but at these conditions the composite already presents a rather high [54] plateau conductivity of 2·10^−6^ S/cm at room temperature. Similarly, it was proposed that the increasing amount of a fully organic IL (1‐ethyl‐3‐methylimidazolium bis(trifluoromethylsulfonyl)imide) in the amorphous phase of PVDF induces the rise of conductivity [55]. At 30 and 40 wt.% IL, the conductivity is even higher (plateau value is 4.5·10^−5^ S/cm at 27°C, Figure S7E), which is consistent with the literature data for PVDF blended with [Emim][TFSI] [53]. However, the most pronounced conductivity increase occurs between 0 and 20 wt.% IL. These results show that the micropores filled with IL contribute to the ion mobility, but the main factor is the presence of a sufficient amount of IL inside the mobile amorphous phase of the polymer matrix.

**FIGURE 5:**
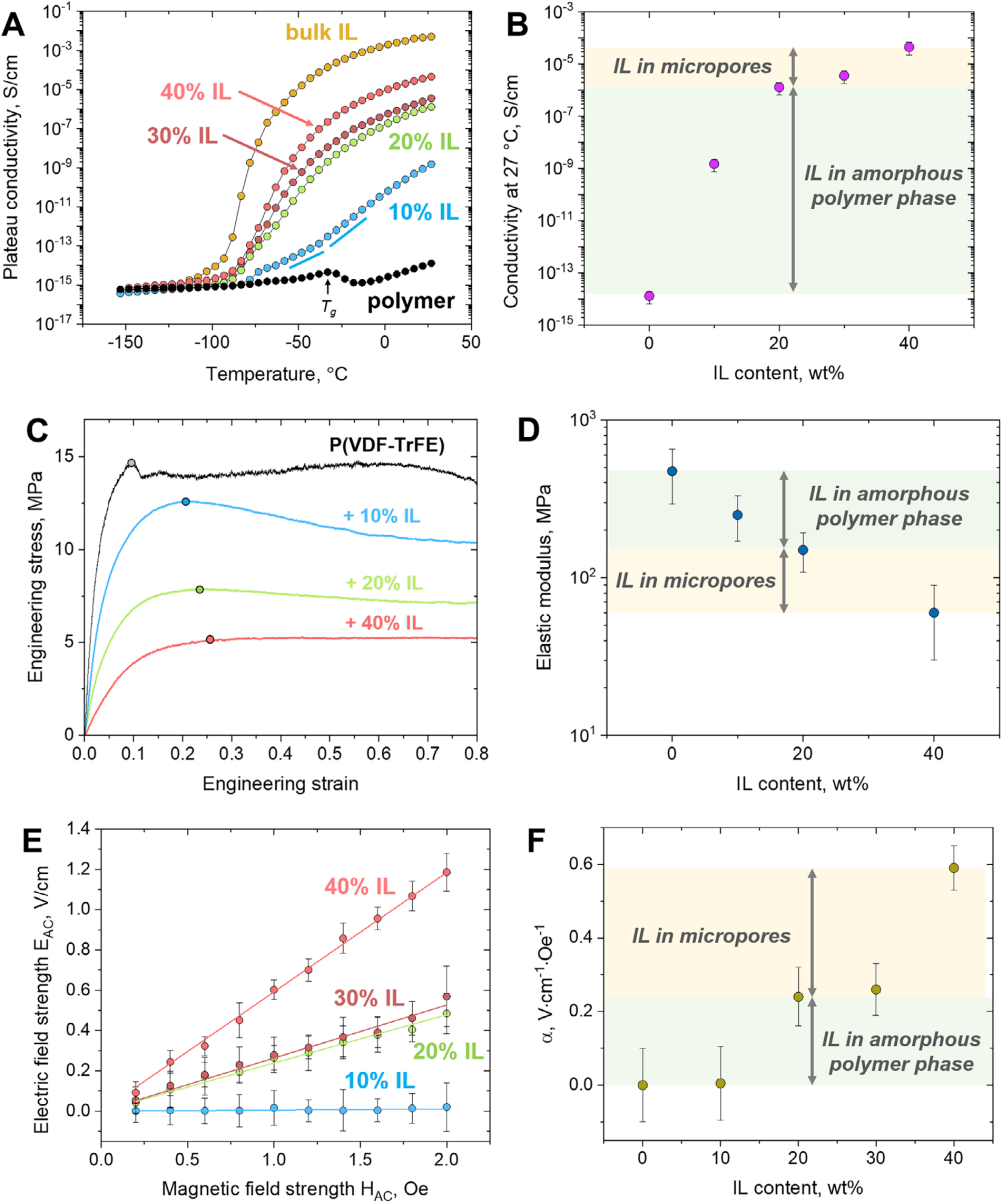
Effect of IL on the macroscopic properties of P(VDF‐TrFE) / [Bmim][FeCl_4_] composites and their components: (A) Temperature dependences of the plateau conductivity (determined from the conductivity frequency dependences, Figure S4; conductivity at 0.05 Hz is taken when the plateau is absent); (B) Plateau conductivity at 27°C as a function of the IL concentration; (C) Tensile stress–strain curves; (D) Dependence of the elastic modulus on the IL concentration; (E) Amplitude of the generated electric field strength (*E_AC_*) vs. amplitude of the applied magnetic field (*H_AC_*) at the frequency of 100 kHz; (F) Magnetoelectric coefficient as a function of the IL concentration.

The above‐described behavior is summarized in Figure 5A, where the plateau conductivities are visualized as functions of temperature. Below the glass transition temperatures of both components (−85°C for the bulk IL, see Figure 1D; and −33°C for P(VDF‐TrFE) [56]), the measured conductivities are extremely low and similar for all the systems, which is reasonable due to the lack of mobility of the IL ions and polymer segments. At higher temperatures, a sharp conductivity increase is observed for all the composites, explained by the onset of the IL mobility. More IL within the composite provides higher conductivity at all temperatures. In the same temperature range, the neat polymer conductivity is low. These data support the fact that the IL is basically responsible for the high ionic conductivity of the composite materials. This effect is similar to the previous reports [53], which, however, did not evaluate the effect of nano‐ and microstructure on conductivity. Note that a slight increase of the σ(*T*) slope is seen for the 10 wt.% IL composite above the polymer *T_g_*, which means that “defreezing” of the polymer segmental mobility also contributes to the increase of conductivity, presumably due to a higher mobility of the IL entrapped in the unconfined amorphous phase. Coupling of the ionic conductivity with polymer segmental dynamics has been observed for several systems, for instance, based on poly(ethylene oxide) [57]. Figure 5B shows the room temperature conductivity as a function of IL concentration. It is seen that the increase of IL content from 0 to 20 wt.% results in the rise of conductivity by nearly 8 orders of magnitude, while changing its concentration from 20 to 40 wt.% induces only a 50‐fold increase of conductivity. This confirms that the incorporation of the IL molecules into the polymer unconfined amorphous phase is the crucial factor determining the conductivity. The IL in the micropores contributes to the rise of conductivity, but not as much as [Bmim][FeCl_4_] intermixed with P(VDF‐TrFE).

The nano‐ and microstructure of the composites also affect their mechanical strength. Two distinct regions are observed in the tensile curves for the neat P(VDF‐TrFE) and its composites with different IL amounts (Figure 5C) – elastic (< ca. 10%–20% deformation) and plastic (higher deformations). An approximate transition between them (i.e. yielding region) is shown by filled circles in Figure 5C. Increasing the IL content has a clear “plasticizing” effect: the stress (exhibited by the composite at each strain) and the elastic modulus (Figure 5D, determined from the initial slope of the tensile curves) decrease, while the regions of linear elastic deformations and plastic deformations are widened. The most pronounced changes occur when the IL content is increased from 0 to 20 wt.%, meaning that the main factor responsible for the “plasticizing” behavior is the incorporation of the IL in the polymer mobile amorphous phase. This aligns with the previous studies, which reported a similar behavior for different ILs incorporated into semi‐crystalline polymer matrices [53, 58]. Note that the formation of micropores occurring at 40 wt.% IL has a weaker effect on the shape of the tensile curves and on the elastic modulus than the IL entrapment in the amorphous phase. However, the decrease of the elastic modulus induced by the IL in the amorphous phase is comparable to the corresponding effect of the micropores (Figure 5D).

In order to demonstrate the influence of the composites´ structure on the properties related to specific device applications, the magnetoelectric performance of polymer/IL materials was evaluated. The magnetoelectric effect is crucial for the operation of magnetic sensing devices [59]. A substantial increase of the generated electric voltage (*V_AC_*) is seen when the frequency of the applied AC magnetic field (with the amplitude *H_AC_* = 2 Oe) exceeds 5 kHz (Figure S8). At a fixed frequency of 100 kHz, the magnetoelectric response is rather strong (Figure S8) and linear vs. magnetic field strength *H_AC_* (Figure 5E). The magnetoelectric coefficient α, calculated as a slope of the dependences of the electric field strength *E_AC_* (*E_AC_* = *V_AC_* / *d*, where *d* is the film thickness) on *H_AC_*, is a “figure of merit” quantifying the magnetoelectric performance. As expected, α increases with the rise of the IL content (Figure 5F), and its values coincide with the data reported for related composites in the same range of frequencies and magnetic field strengths [24]. Several explanations were proposed for the appearance of the magnetoelectric effect. The primary reason is believed to be the polarization of the IL‐filled micropores caused by the movement of the paramagnetic FeCl_4_^−^ ions in the external magnetic field [21]. However, in the work [24], the strongest response was observed for 1‐ethyl‐3‐methylimidazolium tetrachloroferrate(III) / PVDF composite without micropores. In our case, the response of the composite with 10 wt.% IL is most probably beyond the detection sensitivity (it is comparable with the signal detected for the neat polymer matrix, which is likely to arise from the electromagnetic induction at the electrodes). The 20 wt.% IL composite presents only a very small amount of single micropores (Figure 2C); therefore, the detected magnetoelectric response (Figure 5E) arises mostly from the IL embedded into the amorphous phase. Most probably, polarization in the IL nanodomains is already sufficient to create the electric potential. According to the picture of the IL structuration elaborated in this work (Figure 4), the amount of IL in the amorphous phase is the same for the 20, 30, and 40 wt.% composites. At 30 wt.% IL, its excess amount in the micropores is small, and the micropores are small themselves and not interconnected (Figure 2F), leading to a similar value of α as for the 20 wt.% composite. Indeed, magnetoelectric response significantly strengthens with a bigger micropore size [21]. Finally, at 40 wt.% IL the magnetoelectric coefficient is the highest, which is due to the bigger and mostly interconnected pores (Figure 3), containing a significant amount of the IL. Therefore, our results show that the magnetoelectric response is a combined effect of the IL in the amorphous phase and in the micropores.

Additionally, to demonstrate the applicability of the materials as magnetomechanical bending actuators, which is a desirable combination with magnetoelectric response for multifunctional devices [24, 26], bending tests were performed under applied magnetic fields on samples containing different concentrations of magnetic IL. Video S4 shows the configuration of the experiment (details are in Section S11) and demostrates that the film with 30 wt.% IL is bent in the magnetic field of a moving permanent magnet. This effect is explained by the magnetization of the paramagnetic FeCl_4_^−^ ions and their attraction toward the gradient of the non‐uniform magnetic field, which drag the material as a whole and induce the bending deformation. The relative deformation becomes higher with increasing concentration of the IL (Figure S10), since the interaction force between the material and external field is proportional to the total volume of the paramagnetic species (Equation S3).

The correlations revealed in this work show that the macroscopic properties of the materials can be tuned for a particular application by preparing the composites with different structures. For instance, the presence of micropores, important for the magneto‐ionic response, can be achieved by slower solvent evaporation above the polymer melting temperature [21]. By contrast, if saturation of the polymer's amorphous phase with the IL is required without the formation of micropores, the approach presented in this work can be used. It involves rapid solvent evaporation below the polymer's crystallization temperature. As shown above, it allows obtaining the materials with a rather high ionic conductivity while maintaining suitable mechanical properties. A further step in this direction may be related to an independent variation of the micro‐ and nanostructure, which might be achieved by tuning the polymer/IL interactions.

## 3 Conclusions

In this study, we systematically investigated the multiscale structure of P(VDF‐TrFE)/[Bmim][FeCl_4_] composites, and its impact on macroscopic properties critical for applications in sensors, actuators, and energy‐related devices. Combining neutron scattering and cryo‐electron microscopy in a novel methodology, we show that the IL is selectively located within the amorphous regions of the polymer matrix not confined within the crystallites, forming 10–12 nm nanostructures. We identify saturation of the mobile amorphous phase with IL as the decisive factor governing the increase in ionic conductivity, while maintaining sufficient mechanical strength. Crucially, the IL does not penetrate into the crystalline lamellae or into the amorphous phase confined between them, preserving the polymer's degree of crystallinity and the desirable ferroelectric β‐phase. At IL concentrations beyond the saturation threshold of the amorphous phase (30 and 40 wt.%), its excess is microseparated from the polymer matrix into micropores, which provides an additional, though modest, conductivity enhancement. At 40 wt.% of the IL, most of the micropores are interconnected, as revealed by cryo‐electron 3D tomographic reconstruction.

These findings provide a clear strategy for tailoring the internal structure of polymer/IL composites. Fast solvent evaporation below the polymer crystallization temperature enables the incorporation of up to 20 wt.% IL without micropore formation, yielding homogeneous materials with high conductivity and preserved mechanical properties. Conversely, slower solvent evaporation promotes controlled micropore development when such morphology is desired. The results of this work clarify the spatial distribution of IL across nano‐ and microscales and establish a direct correlation with the functional performance of the composites, which is crucial for the advancement of the polymer/IL applications.

Future progress in this field will require extending neutron scattering and electron microscopy studies to systems containing ILs with different chemical architectures and interactions with the polymer matrix. Of particular interest are ILs capable of reducing the polymer crystallinity or intercalating between crystalline lamellae, as these systems may exhibit fundamentally different structure‐property relationships.

## 4 Experimental Section

### 4.1 Materials

Poly(vinylidenefluoride‐*co*‐trifluoroethylene) P(VDF‐TrFE) with a molecular weight *M*_w_ = 350,000 g/mol and a trifluoroethylene monomer content of 30 mol% was provided by Piezotech (Oullins‐Pierre‐Bénite, France). The magnetic IL 1‐butyl‐3‐methylimidazolium tetrachloroferrate(III) ([Bmim][FeCl_4_], purity > 97%) was purchased from Iolitec (Heilbronn, Germany). Acetone (purity > 99%) was obtained from Supelco (Bellefonte, PA, USA).

### 4.2 Film Preparation

The P(VDF‐TrFE) and P(VDF‐TrFE)/IL films were prepared using the solvent casting method. Neat P(VDF‐TrFE) films were obtained by dissolving the polymer (15% w/w) in acetone (85% w/w) at room temperature under magnetic stirring until complete dissolution. The solution was then spread onto a glass substrate, leveled using a doctor blade, and dried in an oven (J.P. Selecta S.A.U., Barcelona, Spain) at 50°C for 15 min. The IL/polymer films (thickness ≈ 50–90 µm) were prepared using a similar procedure; however, as a first step, the IL (10, 20, and 40% w/w with respect to the final sample) was dispersed in the solvent, followed by polymer addition (15% w/w relative to acetone) and subsequent dissolution.

### 4.3 Attenuated Total Reflectance Fourier‐Transform Infrared Spectroscopy

Attenuated total reflectance Fourier‐transform infrared spectroscopy (ATR‐FTIR) was used to analyse the vibrational bands of the polymer and IL in the composites. The measurements were performed using a FT‐IR 6300 instrument (JASCO Corporation, Tokyo, Japan) equipped with a Golden Gate Single Reflection Diamond ATR device, with spectra recorded in the range of 1600 and 500 cm^−1^ at room temperature and a resolution of 4 cm^−1^. Absorption was used to allow quantitative comparison of the band intensities. The data treatment was performed using Essential FTIR software (v.3.5).

### 4.4 Differential Scanning Calorimetry

Differential scanning calorimetry (DSC) was performed using a Q2000 instrument (TA Instruments, New Castle, DE, USA) under ultrapure nitrogen flow. A liquid nitrogen cooling system (LNCS) with a helium flow rate of 25 mL/min was used for temperature control. Samples (2–5 mg) were placed in non‐hermetic aluminium pans and subjected to heating and cooling cycles at a rate of 10°C/min. The measurement protocol included the first heating from room temperature to 180°C, cooling to −150°C, and a second heating to 180°C. Data analysis was performed using TA Universal analysis software (v. 4.5A), including the integration of the Curie transition, cold crystallization, and melting peaks to determine the corresponding transition temperatures.

### 4.5 Scanning Electron Microscopy

Scanning electron microscopy (SEM) was used to examine the microstructure of the composite films. To analyse their cross‐sections, the films were plunged into liquid nitrogen and fractured immediately with tweezers. The cross‐sections were then sputter‐coated with a ≈15 nm gold layer using a K550X sputter coater (Emitech Ltd., Ashford, UK). Imaging was performed with a S‐4800 microscope (Hitachi, Tokyo, Japan) equipped with a cold cathode Field Emission Gun (FEG), operating at 5–7 kV and a working distance of 7–9 mm.

### 4.6 Energy Dispersive X‐Ray Mapping

Energy dispersive X‐ray (EDX) mapping was employed to construct the depth profile of the IL concentration within the composite and was conducted using a JSM‐6390LV scanning electron microscope (JEOL, Tokyo, Japan) equipped with X‐ACT analyzer (Oxford Instruments, Abingdon, UK). A high‐energy electron beam (30 keV) was used to ensure its deep penetration (∼ 12 µm) into the material and the collection of the EDX signal from a bigger volume. The film was fractured in liquid nitrogen, and EDX spectra were collected from 42 slices of the cross‐section, each with a rectangular shape of 2 µm × 80 µm, oriented parallel to the film surface.

### 4.7 Cryo‐Scanning Electron Microscopy/EDX/Cryo‐Tomography

Cryo‐scanning electron microscopy (cryo‐SEM)/EDX/tomography experiments at cryogenic conditions were performed in order to preserve the native structure of the composites. The experiments were performed using an Amber X scanning electron microscope (TESCAN, Brno, Czech Republic) combined with a Xe‐plasma focused ion beam column. Cryo‐conditions were provided by the PP3010 system (Quorum Technologies, Ashford, UK). EDX measurements were performed with an Octane Elect+ detector (EDAX, Mahwah, NJ, USA). Two approaches were used for the sample preparation: (1) freeze‐fracturing of the sample to produce the cross‐sectional view with highly developed morphology emphasizing the local compositional difference; (2) FIB milling at cryogenic conditions to get precise, clean, and flat cross‐sections of the composite structure, suitable for contrast‐based quantification of the IL phase. Sample preparations and all the experiments were performed at −165°C, which is lower than the glass transition temperatures of both the polymer (−33°C [57]) and the IL (−85°C). This allowed preserving the original structure of the specimens.

The images were acquired using a backscattered electron detector to provide strong elemental contrast between the components, employing an accelerating voltage of 5 kV and beam currents of 100 pA–1 nA. To obtain the cryo‐FIB/SEM tomographic series, first, the sample was roughly cut by FIB operated at the beam energy of 30 keV and beam current of 300 nA to prepare the region of interest for the tomographic series acquisition. Final polishing and cutting of the tomographic slices were achieved with lower currents 1–10 nA. The target slice thickness for the ion‐beam tomography was set up to 50 nm, and the total number of slices was around 520. The images were captured with a resolution of 1250 × 1250 pixels, resulting in the voxel size at the 3D‐reconstructions of 50 × 50 × 50 nm^3^.

Avizo software (ThermoFisher, Waltham, MA, USA) was used to combine acquired slices into a 3D volume, and to further perform threshold‐based segmentation (binarization) and analysis. ImageJ software [60] was used to construct pore size histograms, to calculate the pore aspect ratio, and the total pore surface area at the 2D images.

Cryo‐SEM/EDX experiments on the sample with 30 wt.% IL were performed at the Helios NanoLab 650 dual beam microscope (FEI/Thermo Fisher, Hillsboro, OR, USA) equipped with a Ga^+^ FIB column. Cryo‐preparation was performed using a PP3010 cryogenic sample preparation system (Quorum Technologies, Ashford, UK). First, the sample was immersed in liquid nitrogen, fixed in the sample holder by a mixture of polyvinyl alcohol, Tissue‐Tek, and colloidal graphite to ensure rapid freezing and preservation of the native structure under cryogenic conditions, and immediately immersed in liquid nitrogen. The mounted sample was cryo‐fractured, then inserted into the preparation chamber under vacuum, and surface sublimation was carried out for 15–20 min to remove superficial ice contamination. The freshly exposed fractured surface was subsequently sputter‐coated with platinum to improve electrical conductivity and image quality. The sample was then transferred under vacuum to a cryo‐stage of the microscope and examined at −140°C.

### 4.8 Cryogenic Transmission Electron Microscopy/EDX/Tomography

Cryogenic high‐angle annular dark‐field scanning transmission electron microscopy (cryo‐HAADF‐STEM) experiments were performed at 200 kV on a Talos F200i field emission gun instrument (ThermoFisher, Waltham, MA, USA) equipped with a X‐Flash100 XEDS spectrometer (Bruker, Berlin, Germany). Elemental maps were performed by XEDS with a HAADF detector for Z contrast imaging in STEM conditions. The cryo‐sections of about 80 nm thickness were obtained at −90°C using an EM FC6 cryoultramicrotome (Leica, Wetzlar, Germany) equipped with a diamond knife. The sections were placed on 300 mesh copper grids. The frozen grids were then transferred to a 626 DH single tilt cryo‐holder (Gatan, Évry, France), where they were maintained below −170°C (liquid nitrogen temperature) and then were transferred to cryo‐TEM.

3D‐tomographic series were acquired at cryogenic conditions by tilting the sample (± 60°) using a Gatan 914 cryo‐tomography holder. Tilt‐series alignment was performed by an in‐house script using Digital Micrograph (Gatan). 3D‐reconstruction was done by a total variance minimization algorithm realized in Tomviz software [61].

### 4.9 Small‐Angle Neutron Scattering

To investigate the nanoscale structure of the composites, small‐angle neutron scattering (SANS) measurements were performed at the SANS‐I instrument (Paul Scherrer Institute (PSI), Switzerland) [62]. Scattered intensity was measured as a function of the scattering vector magnitude *q* = 4π/*λ* sin(*θ*), where *λ* is the neutron wavelength and *2θ* the scattering angle. Measurements were carried out at a fixed *λ* = 5 Å with two sample‐to‐detector distances (4.5 and 18 m), covering a wide *q*‐range (0.003–0.25 Å^−1^). Raw data were normalized for neutron flux variations, and the intensity at 18 m was converted into the absolute units (cm^−1^) using an H_2_O standard. Primary data treatment included corrections for the sample transmission and subtraction of the background scattering (measured with an empty beam). Scattering length densities (SLDs) of the different parts of the composites were calculated with the NIST SLD calculator software (v. 2.0.0). Fitting of the scattering curves was performed by SasView software (v. 5.0.6). A Lorentz‐corrected plot for the neat polymer was fitted by an asymmetric double sigmoidal function in Origin 2022 software.

### 4.10 Small‐Angle X‐Ray Scattering

SAXS experiments were performed using a 3‐pinhole PSAXS‐L system (Rigaku, Tokyo, Japan) operating at 45 kV and 0.88 mA. The equipment includes a MicroMax‐002 + X‐ray generator, consisting of a microfocus sealed tube source and an integrated Cu K_α_ X‐ray generator unit (photons wavelength λ = 1.5406 Å). Both the flight path and sample chamber are maintained under vacuum. The scattered X‐rays are detected by a hybrid photon counting detector EIGER2 R 1M‐RW (Dectris, Baden‐Dättwil, Switzerland), which provides micrometric spatial resolution (75 µm × 75 µm) over an active area of 77.1 mm × 79.65 mm with 1M pixels. Azimuthally averaged scattered intensities were obtained as a function of the scattering vector *q*. Reciprocal space calibration was carried out using silver behenate as the standard. The samples were measured in transmission geometry with three sample‐to‐detector distances of 2, 0.5, and 0.27 m, covering a *q*‐range of 0.008 to 1.7 Å^−^^1^. At each sample‐to‐detector distance, 9 detector sections were combined with a 3 × 3 Detector Scan Profile (DSP) and with an exposure time of 200 seconds per scan. Temperature‐dependent measurements were conducted using a THMS600 temperature controller (Linkam Scientific Instruments, Tadworth, UK), ensuring a temperature stability of 0.1 K at a stabilization time of 10 min.

### 4.11 Electrical Conductivity

Frequency dependences of the electrical conductivity were measured using an Alpha‐A+ high‐resolution analyzer (Novocontrol, Montabaur, Germany) with an applied AC voltage of 1 V amplitude. The samples were placed between two circular gold‐plated electrodes in a parallel plate capacitor configuration, with diameters of the upper smaller electrode of 10 and 20 mm for films and liquids, respectively. Isothermal scans were obtained in the frequency range between 0.05 and 10^7^ Hz at different temperatures with an interval of 5°C, and the following temperature program was employed: each sample was cooled down from 27°C to −153°C, and then heated back to 27°C. Sample temperature was controlled by exposure to a heated gas stream evaporated from a liquid nitrogen container, provided by a BDS 1100 cryostat.

### 4.12 Tensile Mechanical Tests

Mechanical properties were evaluated by tensile testing using an AGS‐X universal testing machine (Shimadzu, Kyoto, Japan) with 20 × 50 mm plain jaws, vise grips, a 10 mm gauge length, and a 500 N load cell. The uniaxial displacement rate was set to 1 mm/min.

### 4.13 Magnetoionic Response

To characterize the magnetoionic response, gold electrodes with dimensions of 20 mm × 3 mm were first sputtered on both sides of the fabricated films using an EMS/Quorum‐150T‐ES magnetron sputter (Quorum Technologies, Ashford, United Kingdom). Subsequently, electrical wires were glued to each electrode using a conductive silver paste DM‐SIP‐3060HRS (Dycotec, Calne, UK). The contacted samples were placed at the center of a custom‐made Helmholtz coil (3 cm diameter, coil sensitivity *K* = 16.5 Oe∙A^−1^), so that the applied AC magnetic field (*H_AC_*) was oriented in‐plane, i.e., parallel to the film surface. Since the measurements require the application of an AC magnetic field, the dynamic response of the fabricated Helmholtz coils was calibrated. No resonances were observed up to approximately 1.4 MHz, enabling reliable magnetoionic characterization up to 200 kHz. Additionally, both the sample and the coil were placed inside a Faraday cage so that electromagnetic interferences were minimized, thus reducing the measurement noise. A waveform generator Thandar TG‐102 (TTi, Huntingdon, UK) fed the Helmholtz coils, generating an AC magnetic field that was monitored using a gaussmeter GM‐08 (Hirst Magnetic Instruments, Falmouth, United Kingdom). In order to determine the dynamic magnetoelectric response, the excitation frequency was swept between 10 Hz and 200 kHz, while maintaining a constant applied field of 2 Oe amplitude. Then, the dependence of the amplitude of the generated electric field strength (*E_AC_*) on the amplitude of the applied magnetic field strength (*H_AC_*) was studied at a fixed 100 kHz excitation, with applied magnetic fields ranging from 0.2 to 2 Oe. The amplitude of the sinusoidal voltage signal generated by the samples (*V_AC_*) was recorded using an oscilloscope RSDS‐1052DL + (Rigol Technologies, Beijing, China). A small signal from the neat polymer, arising from the induction at the electrodes, was subtracted from all the data. The magnetoionic coefficient, defined as the figure of merit (FoM) of a magnetoelectric device, was calculated as the slope of the dependence *E_AC_* (*H_AC_*) using the following expression:

(4) $$\alpha =\frac{V_{\mathrm{AC}}}{t\cdot H_{\mathrm{AC}}}=\frac{E_{\mathrm{AC}}}{H_{\mathrm{AC}}}$$

where *t* is the film thickness.

### 4.14 Statistical Analysis

The confidence intervals of the measured experimental variables were estimated from statistical errors extracted from several independent measurements, and systematic errors of each experimental method (Section 12, Supporting Information).

## Author Contributions

S.L.M. and A.A. conceived the work. S.L.M., A.A., V.P., and J.M. guided the project. A.S.C. and D.M.C. prepared the samples. A.S. and J.M. performed FTIR, DSC, and mechanical measurements. A.S., J.M., J.M.P., and J.K. conducted the SANS experiments. E.M. and A.Ch. performed the cryo‐SEM/EDX/tomography experiments and analyzed the results, including the 3D‐reconstructions. E.M. partially performed the cryo‐TEM/EDX experiments and data analysis. M.B., L.C., and J.M.T. performed additional cryo‐SEM/EDX experiments. A.I.I. did the SAXS measurements. J.F.M. performed magnetoelectric and bending characterization. A.S. and J.M. performed the conductivity measurements. All authors participated in the discussion and interpretation of the results. A.S., P.S., J.M., and V.P. analyzed the experimental data with contributions from all the authors. A.S. wrote the paper with contributions from all the authors.

## Conflicts of Interest

The authors declare no conflicts of interest.

## Supporting information




**Supporting File 1**: adma72907‐sup7‐00017‐SuppMat.pdf.

**Supporting File 2**: adma72907‐sup‐0002‐VideoS1.wmv.

**Supporting File 3**: adma72907‐sup‐0003‐VideoS2.mp4.

**Supporting File 5**: adma72907‐sup‐0004‐VideoS3.mp4.

**Supporting File 6**: adma72907‐sup‐0005‐VideoS4.mp4.

## Data Availability

The data that support the findings of this study are available upon reasonable request from the authors and in the Supporting Information of this article.
