# Supplementary material for: Resolving Complex Multiscale Structure of Magneto‐ and Electroactive Polymer Composites With an Ionic Liquid
Source: Adv Mater. 2026 Apr 1;38(25):e16835. doi: 10.1002/adma.202516835 (PMC13137764; doi:10.1002/adma.202516835)
Supplement: Supplementary file 1 — Supporting File 1: adma72907‐sup7‐00017‐SuppMat.pdf. [file ADMA-38-e16835-s002.pdf]

Supporting Information

Resolving complex multiscale structure of magneto- and electroactive polymer composites with an ionic liquid

Andrey Shibaev, Jon Maiz*, Viktor Petrenko, Amaia Iturrospe, Josu Fernández Maestu, José María Porro, Mariano Barrado, Laura Casado, Joachim Kohlbrecher, Petr Shvets, Evgeny Modin, Ana Sofia Castro, Andrey Chuvilin, José María De Teresa, Daniela M. Correia, Arantxa Arbe, Senentxu Lanceros-Méndez*

1. Additional DSC data

Table S1. Characteristic transition temperatures and degrees of crystallinity for P(VDF-TrFE) films with different amounts of IL determined from the DSC first heating/cooling cycle.

IL, wt%	T_{Curie}, °C	T_{melt}, °C	T_{cryst}, °C	χ_c, %
neat IL	-	-	-	-
0	99 ± 4	149 ± 2	132 ± 2	23 ± 3
10	101± 4	147 ± 2	130 ± 2	22 ± 3
20	101± 4	147 ± 2	130 ± 2	22 ± 3
30	101± 4	146 ± 2	127 ± 2	22 ± 3
40	101± 4	146 ± 2	128 ± 2	23 ± 3

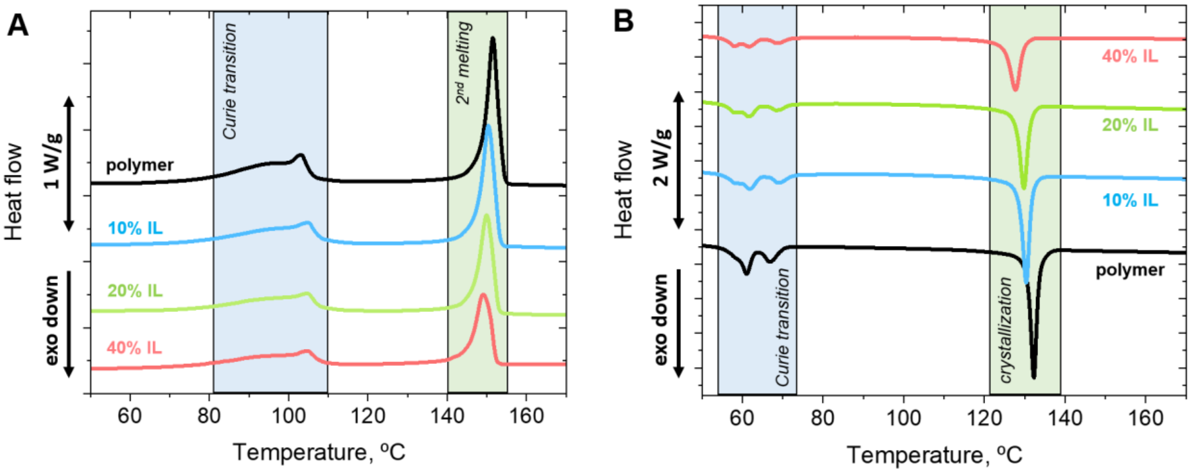

Figure S1. (A) Second heating profile, showing Curie transition and second melting. (B) First cooling profile, highlighting crystallization and Curie transition.

2. Additional cryo-SEM / EDX data

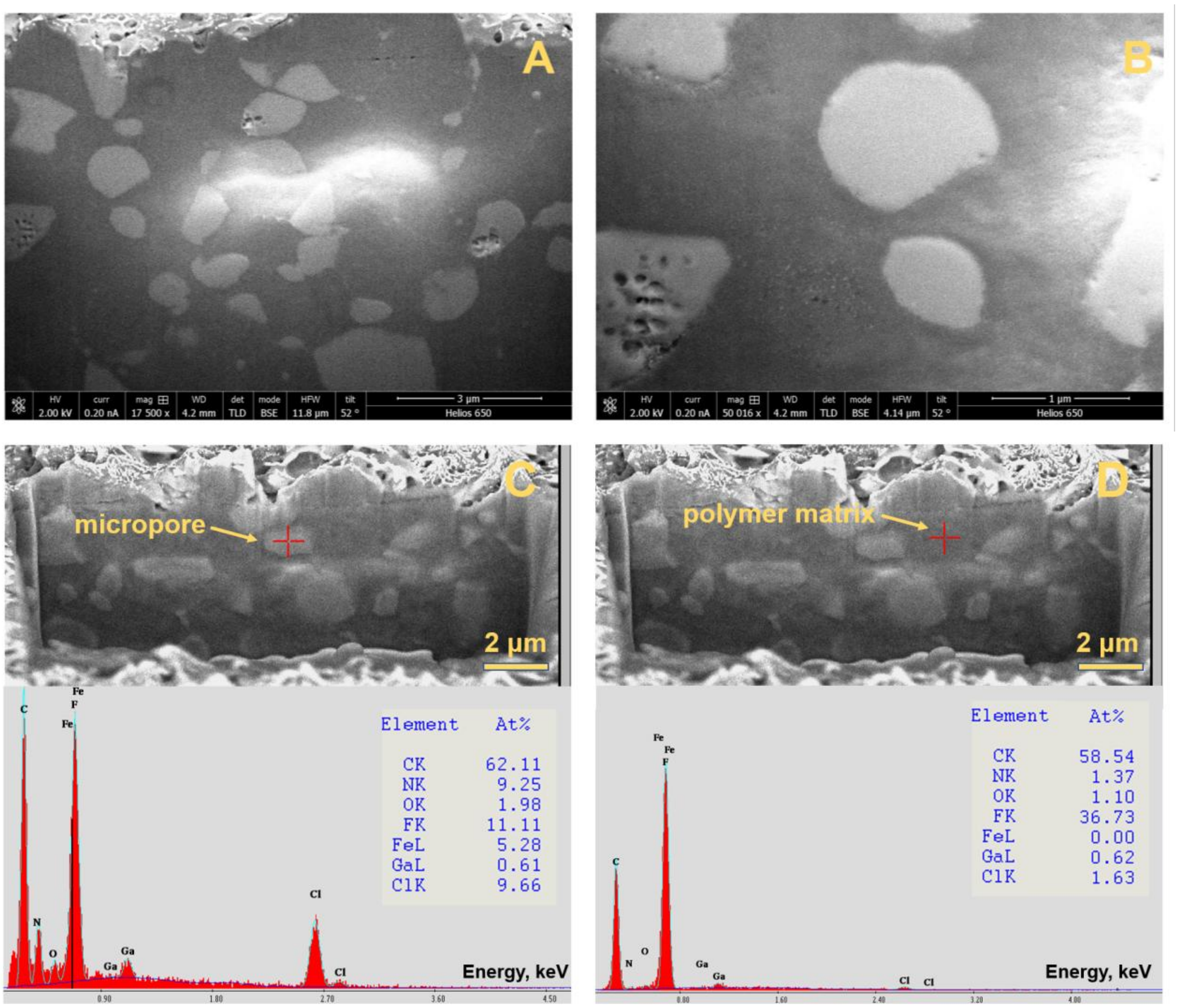

Figure S2. Representative cross-section micrographs obtained by FIB milling for the P(VDF-TrFE) composite with 40 wt% [Bmim][$FeCl_4$], and EDX spectra of the sample areas correponding to the micropore (C) and to the polymer matrix (D). Points of the EDX spectra acquisition are shown by red crosses in the corresponding micrographs.

Some detected F signal in the micropore is most likely Fe line overlapping with the F line. Some F signal may also arise from the limited spatial resolution of EDX spectroscopy and collection of some signal outside of the micropore.

3. Additional cryo-electron tomography data

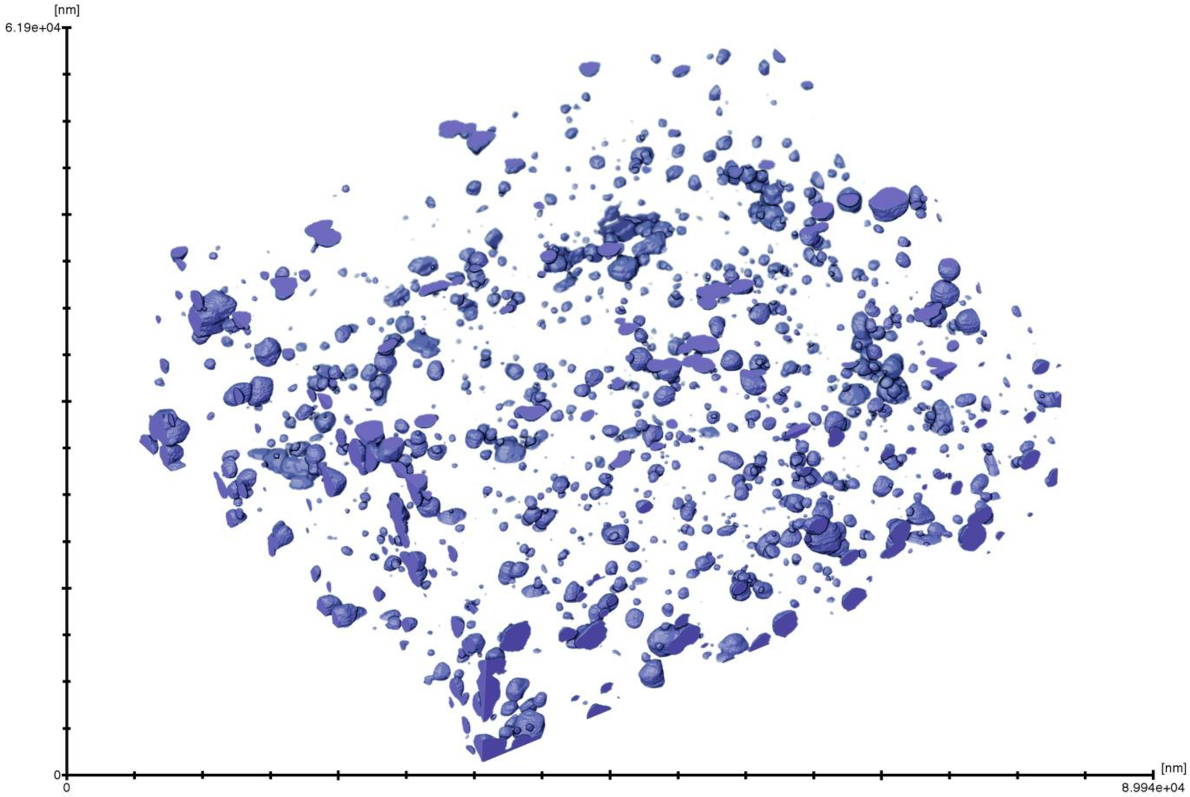

Figure S3. 3D-reconstruction of the non-interconnected micropores from the cryo-FIB/SEM data

4. Gravimetric densities and SLDs

Table S2. Gravimetric densities and SLDs of the different parts of the composites

**Part of the composite**	**Gravimetric density, g/cm^3**	**neutrons SLD, 10^{-6} Å^{-2}**
polymer<br>crystalline ß-phase	1.97 [a]	3.4 [c]
polymer<br>amorphous phase	1.68 [a]	2.9 [c]
ionic liquid<br>[Bmim][$FeCl_4$]	1.36 [b]	1.5 [c]

[a] Meng N., et al. Nature Communications 2019, 10, 4535
[b] According to the manufacturer
[c] Obtained with the NIST SLD calculator (https://www.ncnr.nist.gov/resources/activation/)

5. Fitting of the neutron scattering curves

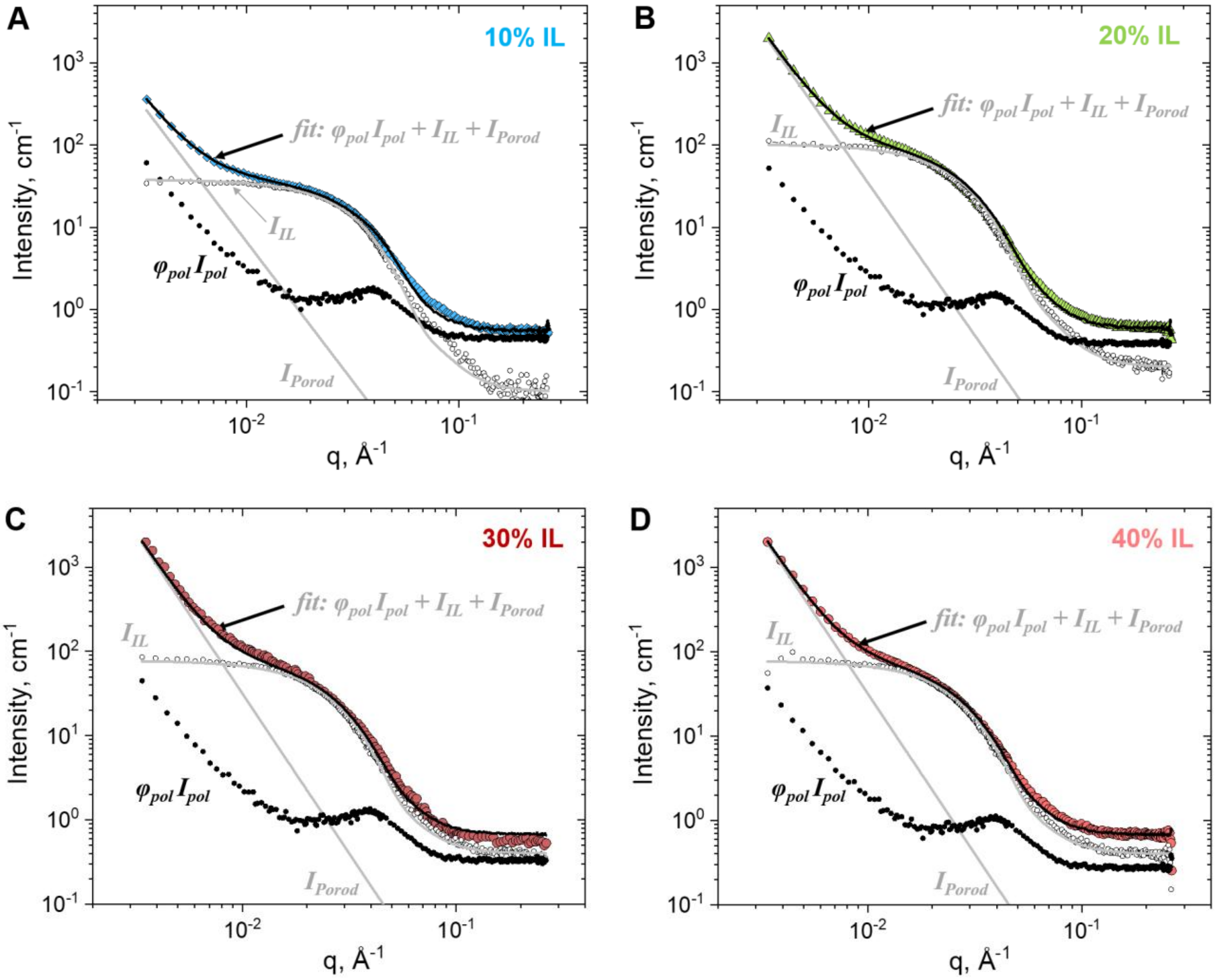

Figure S4. Decomposition of the neutron scattering curves into three contributions – Porod scattering from the surfaces of large objects at low q, scattering form the polymer matrix, and scattering form the IL nano-accumulations inside the polymer matrix:

- experimental neutron scattering curves for the composites at different IL concentrations (blue, green and red symbols);

- grey solid lines I_{Porod} fit the slope of the scattering curves at low q-s;

- $\varphi_{pol}I_{pol}$ (black circles) is the experimental scattering curve of the polymer matrix normalized by the volume fraction of polymer in the composites;

- open circles are the results of subtraction of I_{Porod} and $\varphi_{pol}I_{pol}$ from the experimental scattering curves of the composites, representing the form-factor of IL nanostructures;

- I_{IL} (grey lines) are fits of the form-factors of IL nanostructures by a model of a polydisperse sphere (fitting parameters are presented in Table S3);

- black lines are the total fits of the experimental scattering curves of the composites, which are sums of three contributions: $I_{Porod} + \varphi_{pol}I_{pol} + I_{IL}$

Table S3. Characteristics of the P(VDF-TrFE)/[Bmim][$FeCl_4$] composites derived from SANS

IL, wt%	Porod exponent *P*	Polymer lamellae long period L_c, nm	Diameter of the IL nano-accumulations, nm	Diameter polydispersity [b]
0	3.1 ± 0.3	14 ± 0.4	-	-
10	3.4 ± 0.5	14 [a]	10.2 ± 0.5	0.30 ± 0.10
20	3.7 ± 0.4	14 [a]	11.2 ± 0.6	0.35 ± 0.10
30	3.8 ± 0.4	14 [a]	11.6 ± 0.6	0.35 ± 0.10
40	3.8 ± 0.4	14 [a]	11.6 ± 0.6	0.35 ± 0.10

[a] *L_c is assumed to be the same as for neat P(VDF-TrFE) due to the constant degree of crystallinity χ_c*

[b] *polydispersity of log-normal size distribution (standard deviation of the logarithm of the size*

6. Temperature-dependent SAXS data

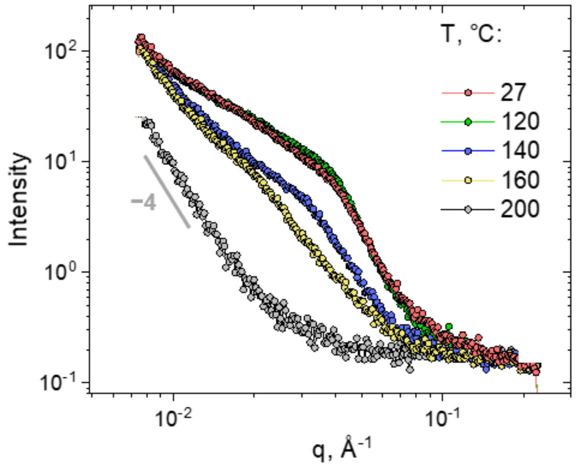

Figure S5. SAXS scattering curves for P(VDF-TrFE) composite with 40 wt% IL at different temperatures.

7. Calculation of the relative volume of micropores

Let us consider a P(VDF-TrFE) composite with a total of 40 wt% [Bmim][$FeCl_4$] IL, and let us assume a simple case that all the IL is located in the micropores. The total volume of the micropores relative to the whole volume of the composite is:

$$\frac{V_{pores}}{V_{tot}} = \frac{\frac{m_{IL}}{\rho_{IL}}}{\frac{m_{IL}}{\rho_{IL}} + \frac{m_{pol}}{\rho_{pol}}} = \frac{\frac{0.4\ g}{1.36\ g/cm^3}}{\frac{0.4\ g}{1.36\ g/cm^3} + \frac{0.6\ g}{1.8g\ /cm^3}} = 0.47 \qquad \text{(S1)}$$

Let us consider a more complex case, when half of the IL resides in the micropores, and half is located in the polymer matrix. In this case, the relative volume of the micropores is:

$$\frac{V_{pores}}{V_{tot}} = \frac{\frac{m_{IL}/2}{\rho_{IL}}}{\frac{m_{IL}}{\rho_{IL}} + \frac{m_{pol}}{\rho_{pol}}} = 0.24 \qquad \text{(S2)}$$

8. Depth distribution of the IL

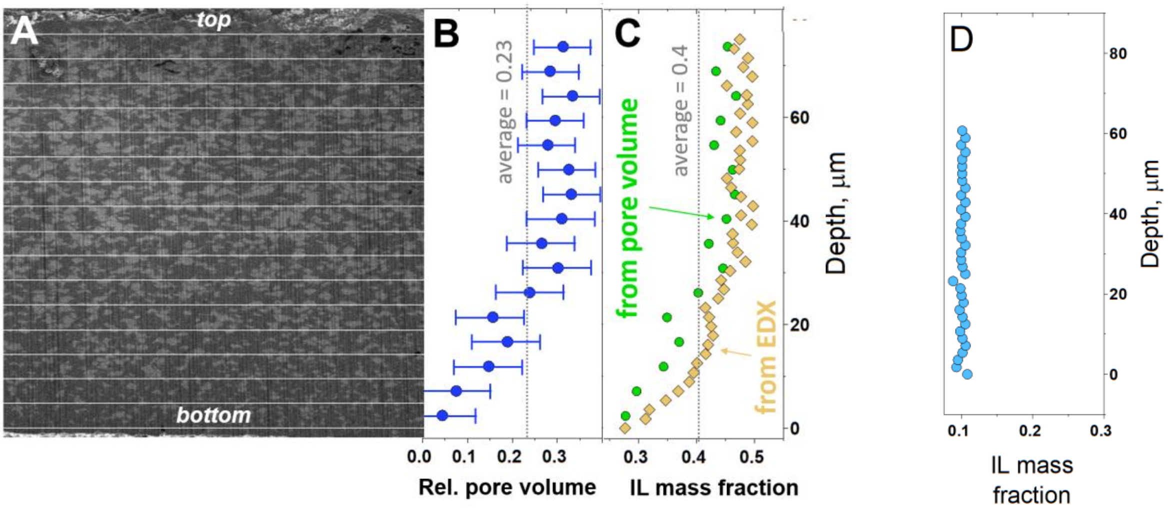

Figure S6. Depth profiling of the P(VDF-TrFE) composite containing 40 wt% [Bmim][$FeCl_4$]: (A) Cross-section cryo-SEM micrograph obtained by FIB milling, divided into 16 slices. (B) Volume fraction of the micropores vs depth (calculated using Equation 3). (C) IL concentration (w/w) vs depth calculated by analysis of the volume of micropores (green symbols) and obtained by EDX mapping (taken from another cross-section, orange symbols). (D) IL concentration calculated obtained by EDX mapping for 10 wt% IL composite

In order to evaluate the depth profile of the IL concentration, the composite cross-sectional image (from FIB milling and cryo-SEM) was divided from bottom to top into 16 slices with a thickness of 4.75 µm (**Figure S6**). The relative pore volume fraction was estimated within each slice using the Equation 3 and plotted in Figure 5B. Indeed, the depth distribution of the pore volume fraction is not uniform, and the bottom layer is poor in pores, consistent with the SEM data (Figure 2D).

From the relative volume of pores (Figure S6), the depth profile of the IL concentration is calculated (Figure S6), assuming that: 1) all the micropores are filled with the IL, 2) half of the IL, i.e. 20 wt%, is uniformly dispersed in the polymer matrix, independent of height. The calculated profile coincides with the depth dependence of the IL concentration obtained from EDX elemental mapping of Cl (IL) and F (polymer). Both methods show that the bottom of the composite has a lower concentration of the IL than the other parts. However, it is seen from Figure 5C that the IL mass fraction does not go below 20 wt% at any depth. This is because the total IL mass fraction at each depth is composed by two contributions: 1) IL in the amorphous phase of the polymer matrix, which is considered to be equal to the saturation concentration of 20 wt%; 2) IL in the micropores. This fact is important for the macroscopic properties: as shown

below, the key factor for a high ionic conductivity of the composites is the saturation of the amorphous phase with the IL. Therefore, even if the micropores are not homogeneously distributed within the sample, the fraction of the IL in the polymer phase would ensure the conductivity.

The presence of micropores is important for some macroscopic properties, for instance, magnetoelectric response. Also, as shown below, the pores contribute to the increase of conductivity, which is expected to be more pronounced if the pores are interconnected. Electron tomography data (Figure 3E) show that the pores are interconnected and seem to be on the onset of percolation (Figure 3G). However, the tomogram was reconstructed from a top part of the film, rich in IL. One can expect that the scarce pores in the bottom part are not interconnected, which may influence the conductivity of the film as a whole.

9. Conductivity data

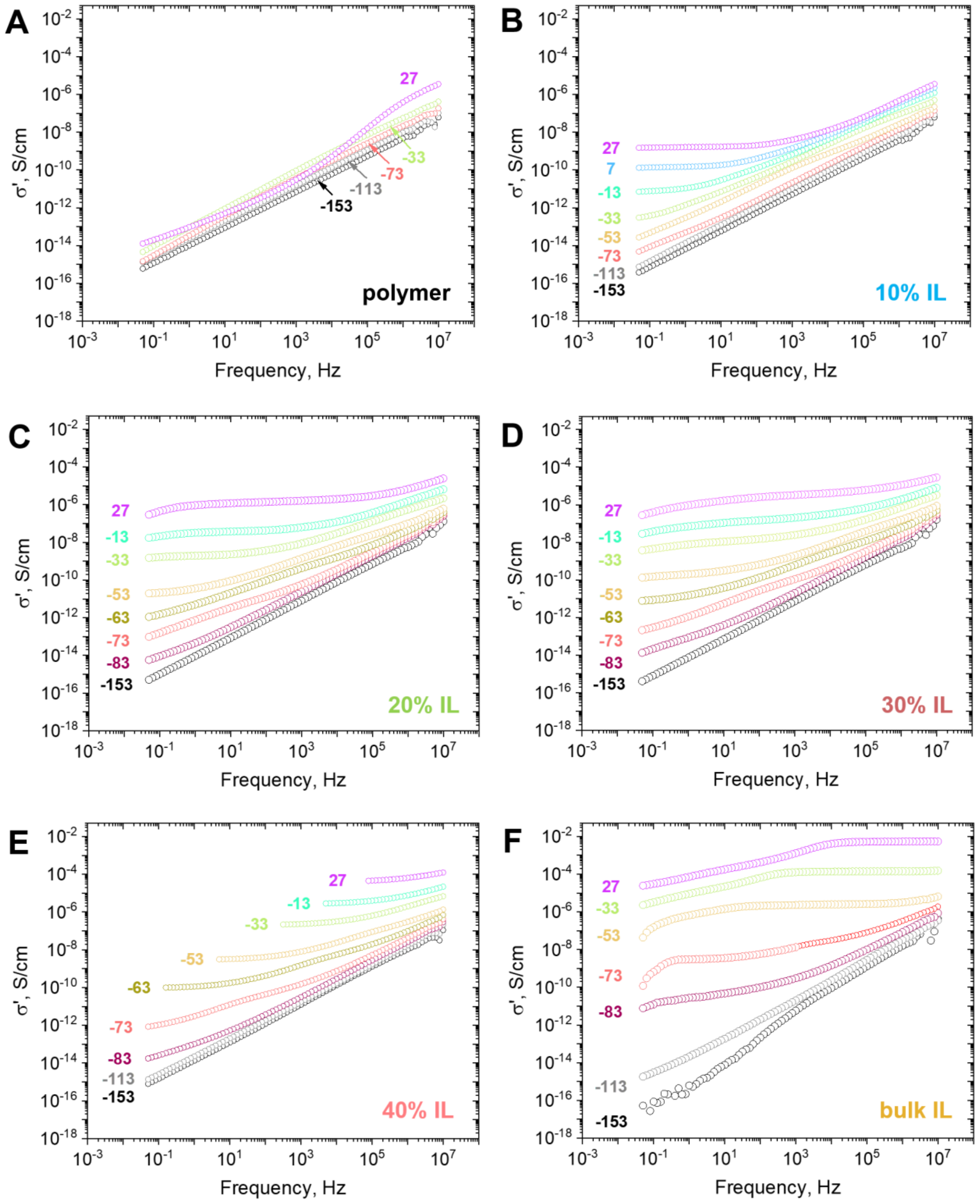

Figure S7. Frequency dependences of the real part of the electrical conductivity at different temperatures (indicated in the Figs in ºC) for P(VDF-TrFE) in the absence of IL (A); in the presence of 10 ~~wt%~~ (B), 20 ~~wt%~~ (C), 30 (D) and 40 wt% IL (E); and for the bulk IL (F).

10. Magnetoelectric response

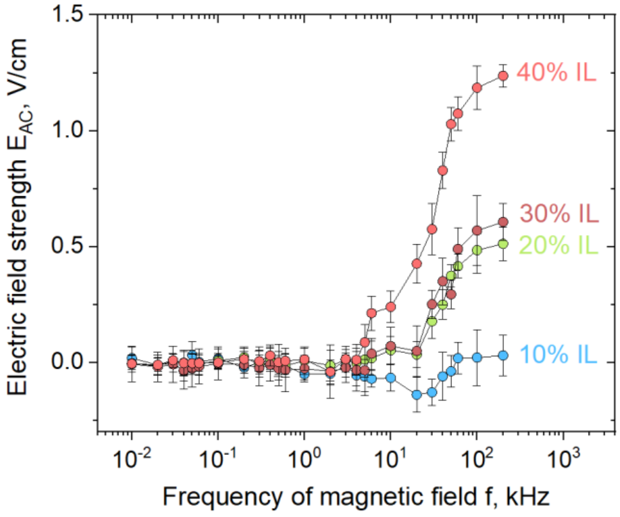

Figure S8. Dependences of the generated electric field strength on the frequency of applied magnetic field (with amplitude H_{AC} = 2 Oe) for P(VDF-TrFE) composites with 10 (blue), 20 (green), 30 (dark red) and 40 wt% IL (red).

11. Magnetic actuation

In order to evaluate the magnetomechanical bending response, the films were clamped with two needles and submitted to magnetic stimulation via a permanent NdFeB magnet (B-30-30-15, N45, Superimanes) attached to a mechanical vibrator (SF-9324, PASCO Scientific). The vibrator was driven sinusoidally at a frequency of 0.1 Hz with a function generator (PI-9598 from PASCO Scientific). This configuration generated a magnetic field varying from a minimum of 45 mT (magnet positioned far from the sample) to a maximum of 175 mT (magnet positioned close to the sample).

Sample deflection (δ) was recorded using the digital camera of a Google Pixel 8a smartphone (64 MP, f/1.89), with 8x digital zoom and 16MP resolution. Video frames corresponding to different applied magnetic fields were subsequently extracted using MATLAB and analysed with ImageJ software, allowing the determination of sample deflection versus time.

The magnetic (Kelvin) force acting on the magnetic composite can be expressed as [Rosensweig, R. E. Ferrohydrodynamics. Cambridge University Press, 1985]:

$$\vec{F} = \int_V \frac{\chi}{2\mu_0} \nabla(B^2)dV \qquad \text{(S3)}$$

where B is the external magnetic field flux density, χ is the magnetic susceptibility of unit volume of the material, μ_0 is the permeability of free space, and the integration is over the volume of the composite subjected to the magnetic field.

Thus, the magnetic force depends on the gradient of B^2, but not on the field itself, so the material deforms only in spatially non-uniform fields. It should be noted that, when a permanent magnet is used, $\nabla(B^2)$ is also non-uniform at the different points of the sample; however, for the purpose of comparison of different samples, we estimate their relative deformations as functions of the mean $\nabla(B^2)$.

The sample deflections were corrected for the sample thickness (t). Since force density is per volume (Equation S3), the total magnetic force acting on the films with the same surface area scales with thickness as

$$F \sim t \tag{S4}$$

The bending modulus for the polymer films scales as [A.P. Boresi, R.J. Schmidt, O.M. Sidebottom. Advanced Mechanics of Materials. 4th Edition, John Wiley & Sons, 1985]

$$D \sim Et^3 \tag{S5}$$

where E is the Young´s modulus. For small film deflections δ under distributed load

$$\delta \sim \frac{F}{D} \sim t^{-2} \tag{S6}$$

To demonstrate this behaviour for our materials, bending of the films was modeled using Comsol Multiphysics software (version 6.1). The film had a shape of simple block with width of 5 mm, length (l) of 40 mm and variable thickness. As an example, the material had density of 1870 kg/m^3, Young's modulus of 100 MPa and Poisson's ratio of 0.35. In order to simulate the experimental conditions, the upper side of the film was fixed, it was placed in a uniform gravity field, and an external magnetic force (F = 232 N/m^3) was applied in a horizontal direction to the bottom half of the film. A default mesh with 'fine' element size was used to compute the deformation. Modelling confirms that deflection is inversely proportional to the squared thickness (Figure S9), as predicted by Equation S6.

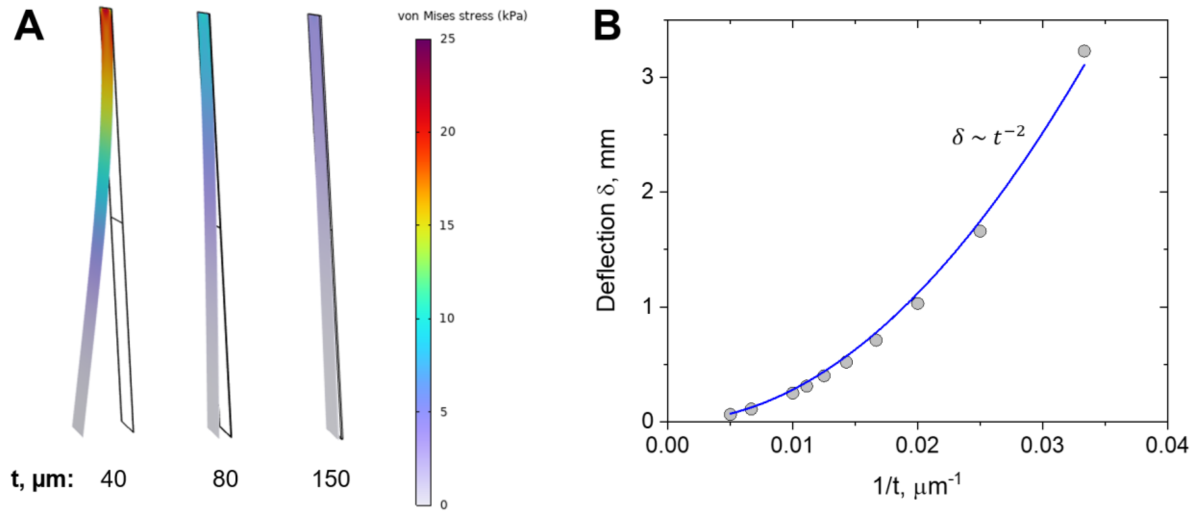

Figure S9. (A) Snapshots of deformation of the films with three different thicknesses (t) in the same external field; (B) Dependence of the deflection of the film lowest point on thickness.

Therefore, the experimentally measured deflections were normalized by the sample free length l and by the square of the sample thickness (relative to a reference thickness of 70 μm), and the normalized relative deformations were calculated as

$$\gamma = \frac{\delta}{l}\left(\frac{t}{70}\right)^2 \cdot 100\% \qquad \text{(S7)}$$

The dependences of γ on $\nabla(B^2)$ are shown in Figure S10 for different IL concentrations.

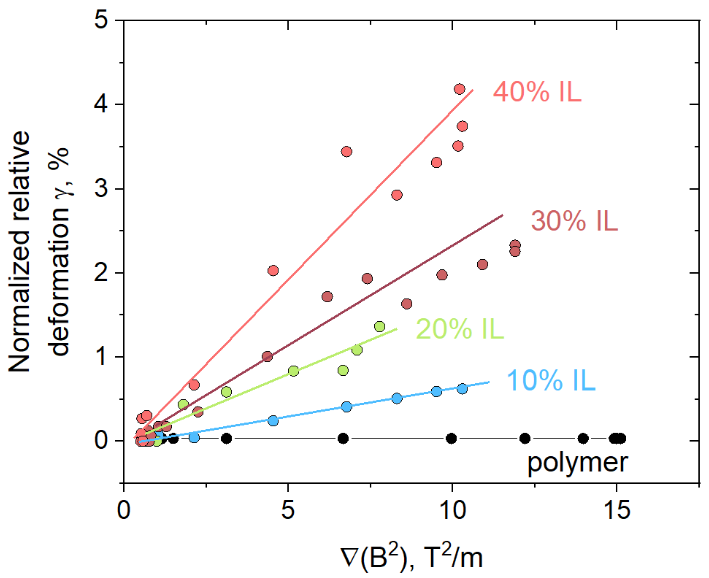

Figure S10. Normalized relative deformations as a function of the gradient of the squared flux density of the external magnetic field for P(VDF-TrFE) film composites with various IL concentrations

12. Estimation of the experimental errors

The confidence intervals (Δx) of the obtained experimental variables were calculated by taking into account the statistical errors σ_{stat}, estimated from N independent measurements of the reproduced samples, and systematic errors σ_{method} of each experimental method.

Let us first consider statistical errors. The mean value of N independent measurements x_i is

$$\bar{x} = \frac{1}{N}\sum_{i=1}^{N} x_i \tag{S8}$$

Standard deviation shows the spread of the individual measurements around the mean:

$$SD = \sqrt{\frac{1}{N-1}\sum_{i=1}^{N}(x_i - \bar{x})^2} \tag{S9}$$

A corresponding statistical experimental error is [Greenland, S., et al. Statistical tests, P values, confidence intervals, and power: a guide to misinterpretations. Eur. J. Epidemiol. 31 (2016), 337–350]

$$\sigma_{stat} = t\frac{SD}{\sqrt{N}} \tag{S10}$$

where t is the Student´s coefficient (critical value of the Student’s t-distribution corresponding to the chosen confidence level). Including systematic errors gives the following equation for the total (statistical and systematic) errors

$$\sigma = \sqrt{\sigma_{stat}^2 + \sigma_{method}^2} \tag{S11}$$

Finally, the confidence interval is expressed by the equation

$$CI = \bar{x} \pm \sigma \tag{S12}$$
